# Supplementary material for: Breakdown of semiclassical description of thermoelectricity in near-magic angle twisted bilayer graphene
Source: Nat Commun. 2022 Mar 21;13:1522. doi: 10.1038/s41467-022-29198-4 (PMC8938509; doi:10.1038/s41467-022-29198-4)
Supplement: Supplementary file 1 — Supplementary Information [file 41467_2022_29198_MOESM1_ESM.pdf]

Supplementary information: Breakdown of semiclassical description of thermoelectricity in near-magic angle twisted bilayer graphene

Bhaskar Ghawri,[1,*] Phanibhusan S. Mahapatra,[1,†] Manjari Garg,[2,‡] Shinjan Mandal,[1] Saisab Bhowmik,[2] Aditya Jayaraman,[1] Radhika Soni,[2] Kenji Watanabe,[3] Takashi Taniguchi,[4] H. R. Krishnamurthy,[1] Manish Jain,[1] Sumilan Banerjee,[1] U. Chandni,[2] and Arindam Ghosh[1,5,§]

[1]*Department of Physics, Indian Institute of Science, Bangalore, 560012, India*
[2]*Department of Instrumentation and Applied Physics, Indian Institute of Science, Bangalore, 560012, India*
[3]*Research center for Functional Materials, National Institute for Materials Science, Namiki 1-1, Tsukuba, Ibaraki 305-0044, Japan*
[4]*International center for Materials Nanoarchitectonics, National Institute for Materials Science, Namiki 1-1, Tsukuba, Ibaraki 305-0044, Japan*
[5]*Centre for Nano Science and Engineering, Indian Institute of Science, Bangalore 560 012, India*

Supplementary Note 1: Device fabrication

In this study, we have fabricated the devices in two different configurations. In the first configuration (denoted by H1) two graphene layers are aligned at an angle θ and encapsulated within two hexagonal boron nitride (hBN) (Supplementary Fig. 1(a)). The device is etched into a hall bar and consists of both single layer graphene (SLG) and twisted bilayer graphene (tBlG) regions. A local gold top gate is fabricated only on the tBLG region, while the global silicon backgate is used to tune the number density (n) in SLG region. The device schematic and optical micrograph in this geometry are shown in Supplementary Fig. 1(b), (c). In the second configuration (denoted by C1), the tBLG device consists of two graphene layers aligned at $60° + \theta$, thus θ being the effective twist angle [1], and encapsulated within two sheets of hBN (see schematic shown in Supplementary Fig. 1(d)). The moiré super-lattice is formed at the overlap region ($\approx 5\ \mu$m $\times$ 6 μm), and the monolayer branches of graphene on four sides act as electrical leads. The device micrograph is shown in Supplementary Fig. 1(e). A local top-gate tunes n of the overlap region. Supplementary Table 1 gives the details of all the different devices used in this study.

We have used Raman spectroscopy to qualitatively differentiate between large and small twist angle tBLG devices and to avoid making devices on tBLG stacks which turn into bilayer graphene (BLG) after the transfer process. Specifically, the shape and position of the $2D$ peak is sensitive to the twist angle and provides a rough estimation[2–4]. Supplementary Fig. 1(f) shows the Raman spectra of $\sim 1.6°$, $\sim 2.4°$, $\approx 4°$ tBLG devices and single layer graphene (SLG). $2D$ peaks of small twist angle devices have higher values of the full width half maximum (FWHM) than SLG and usually have an additional shoulder. In contrast, large twist angle tBLG has a spectrum very similar to SLG as the two layers are decoupled. Though the Raman spectrum does not have enough resolution to precisely determine the twist angle, it provides a qualitative information about the angle. We have used transport methods to calculate the exact twist angle for all devices except D5 ($\theta \sim 4°$), where Raman spectrum is used to estimate the approximate angle.

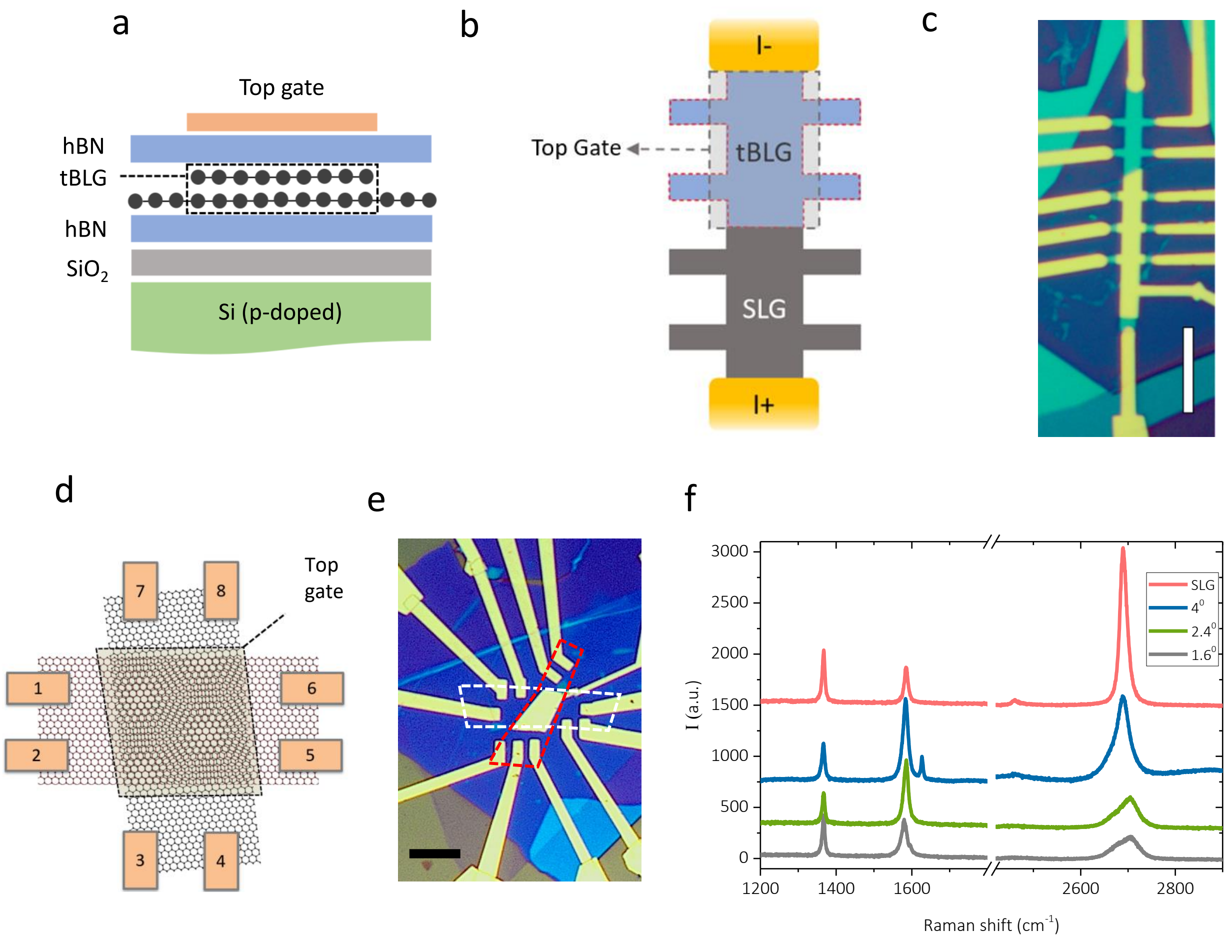

Supplementary Fig. 1: Device fabrication and characterization. (a) Schematic of the cross-sectional view of the device showing the constituent layers. Devices were fabricated in two different geometries, denoted by H1 and C1. (b) Schematic of the device fabricated in hall bar geometry (H1). The dashed rectangle shows the tBLG region covered with gold top gate while the gray region represents the SLG region. (c) Optical micrograph of a typical tBLG device fabricated in H1 geometry. The scale bar represents a length of 5 μm. (d) Device schematic representing the cross-bar geometry (C1). The dashed region shows the tBLG region covered with top gate (e) Optical micrograph of a tBLG device fabricated in G1 geometry. The scale bar represents a length of 5 μm and dotted lines show the graphene edges. (f) Comparison of Raman spectra for G peak and 2D peak obtained for $\approx 1.6°$, $\sim 2.4°$, $\approx 4°$ and single layer graphene with relative offset in the intensity for clarity.

Supplementary Note 2: Experimental details of the different devices in this study

Device name	Twist angle (deg)	Device type
D1	1.01	H1
D2	1.24	H1
D3	1.6	C1
D4	1.7	H1
D5	~ 4	C1

Supplementary Table 1: Details of the devices measured.

Supplementary Note 3. Quantum oscillations in D2 and D3 devices

Supplementary Fig. 2(a) shows the resistance (R) measured at 5 K in the absence of an external magnetic field, and Supplementary Fig. 2(b) shows the results of quantum oscillations measurements in 1.6° device . We have plotted the first derivative of R with respect to the gate voltage tuned from Dirac point ($V_{tg} - V_D$) in order to get a better colour contrast. The quantum oscillations emerging from the CNP are eight fold degenerate as previously reported for devices with similar twist angles[5]. In contrast, the Landau fans emerging from $\nu = \pm 4$ are four fold degenerate, accounting for spin and Fermi-contour degeneracy. Additionally, we have estimated the twist angle using the quantum oscillations, which comes out to be $\approx 1.6°$

Supplementary Fig. 3(a) shows the derivative of the resistance (dR/dV_g) versus gate voltage and $B_\perp$ in device D2. We plot the Landau level structure deduced from the oscillations in Supplementary Fig. 3(b). At low field, The black Landau fan, which originates from the charge neutrality point (CNP), and the pink Landau fan, which originates from the superlattice density, show the filling-factor sequences $\pm 4, \pm 8 \ldots$ as expected for single-particle band structure with four-fold spin and valley degeneracies. We note that as the magnetic field is increased above 5 T, either valley or spin degeneracy breaks and two faint lines with filling-factor ± 2 appear to emerge from the CNP. Additionally, we find multiple other Landau fans (purple lines) emerging from intermediate densities and are not expected from single-particle band structure. The origin of these lines can be traced back to the resistive peaks observed near integer fillings as shown in Supplementary Fig. 3(c)

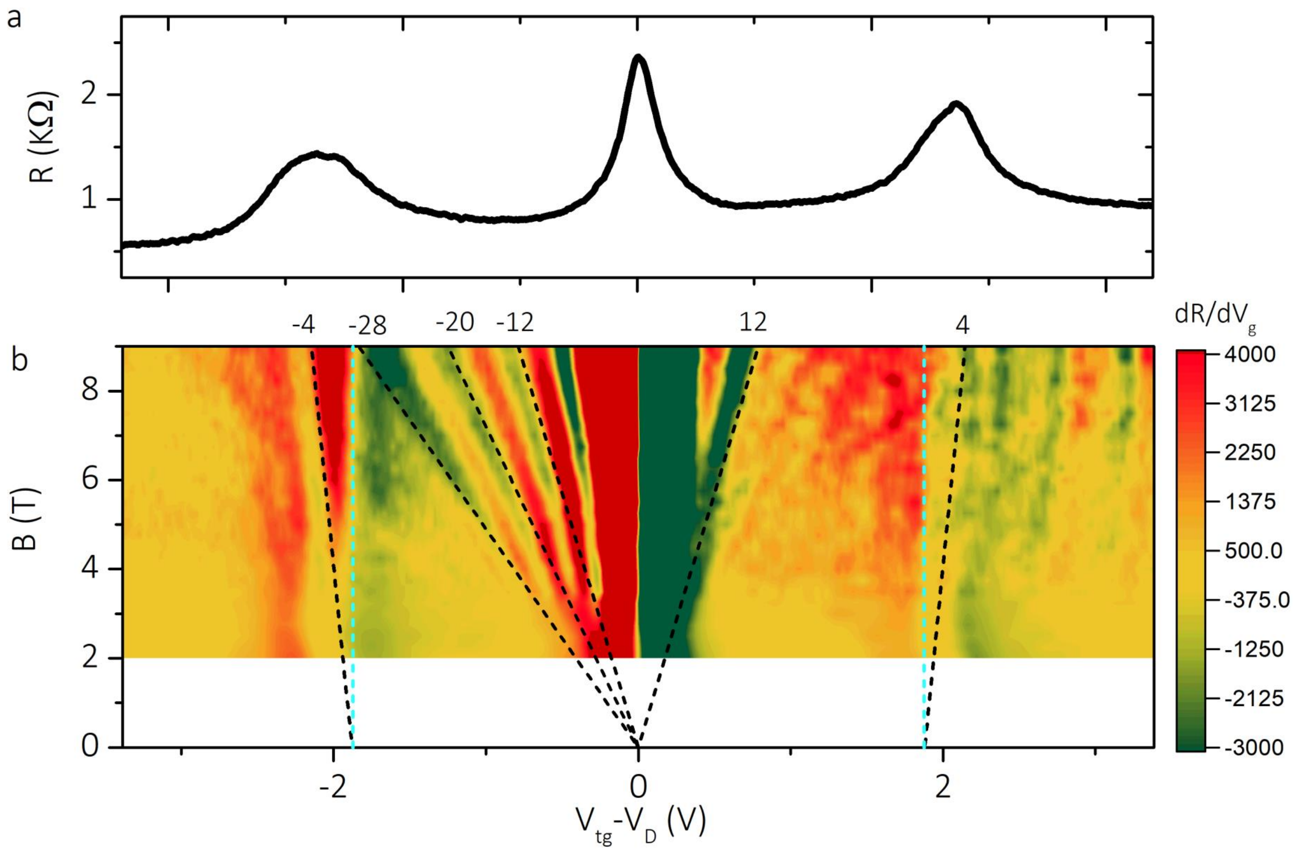

Supplementary Fig. 2: Landau fan in device D3. (a) R as a function of n measured at 5 K. (b) Quantum oscillations in 1.6° device. The first derivative of R with respect to gate voltage is plotted in order to enhance the colour contrast. Black dashed lines show the Landau fans emerging from the CNP and $\nu = \pm 4$.

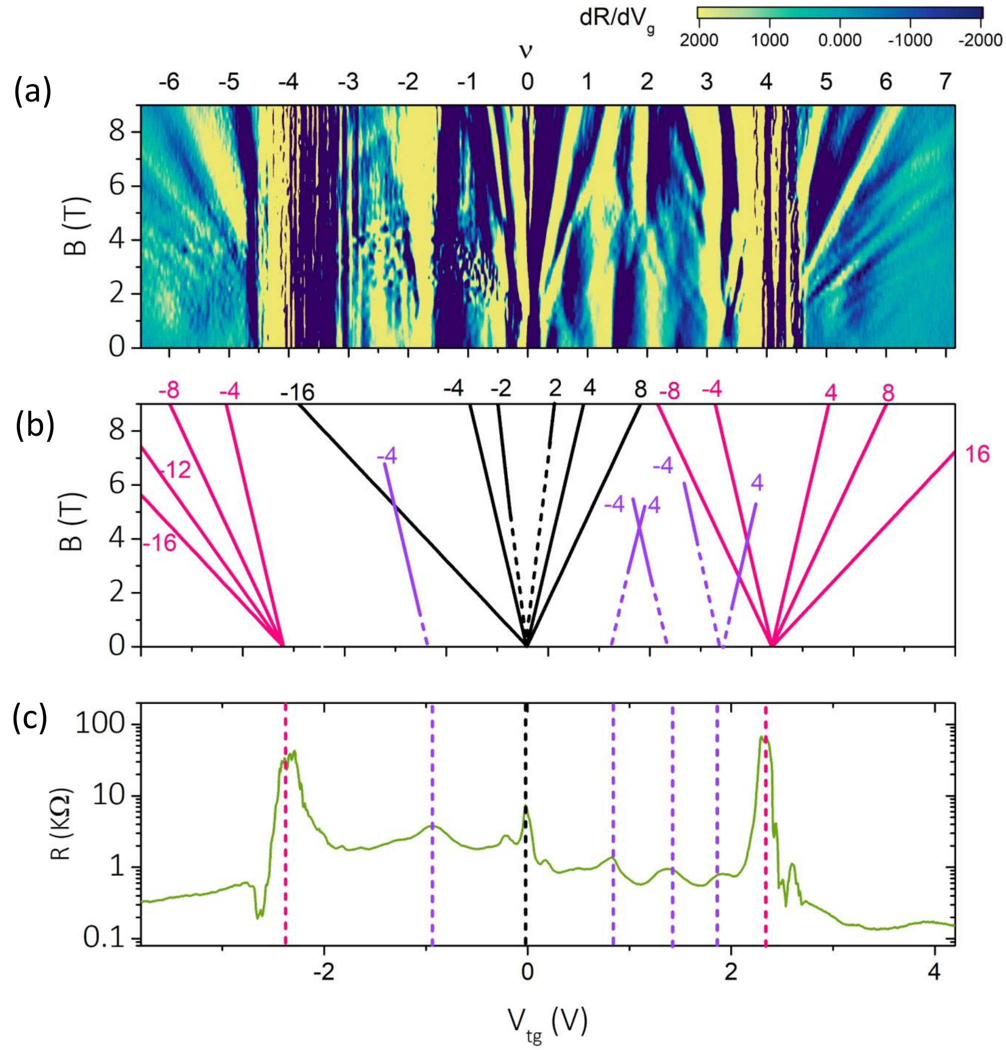

Supplementary Fig. 3: Landau fan in device D2. (a) Quantum oscillations in device D2 (1.24°) measured at 100 mK. (b) Black and pink solid lines show the Landau fans emerging from the CNP and $\nu = \pm 4$, whereas purple lines show fans originating from intermediate fillings, where peaks in R are observed . (c) R as a function of V_{tg} at zero magnetic field. Dashed lines corresponds to resistive peaks which gives rise to Landau fans.

Supplementary Note 4: Additional data on electrical transport in different devices

A. Device geometry and transport measurements in device D1

Device D1 was fabricated with a multilayer WSe_2 between the top hBN and the tBLG (Supplementary Fig. 4 (a)), and the optical micrograph of the device is shown in Supplementary Fig. 4(b). We observe weak resistive features at $\nu = 1, 2$ and 3 at 5 K, which persist upto $20 - 25$ K (denoted by CS) and show a clear metallic behaviour (Fig. 1a). Additionally resistance peak at $\nu = 9$ (Supplementary Fig. 4 (c)) indicates the presence of correlations even in higher bands which have not been studied extensively so far and requires further investigation.

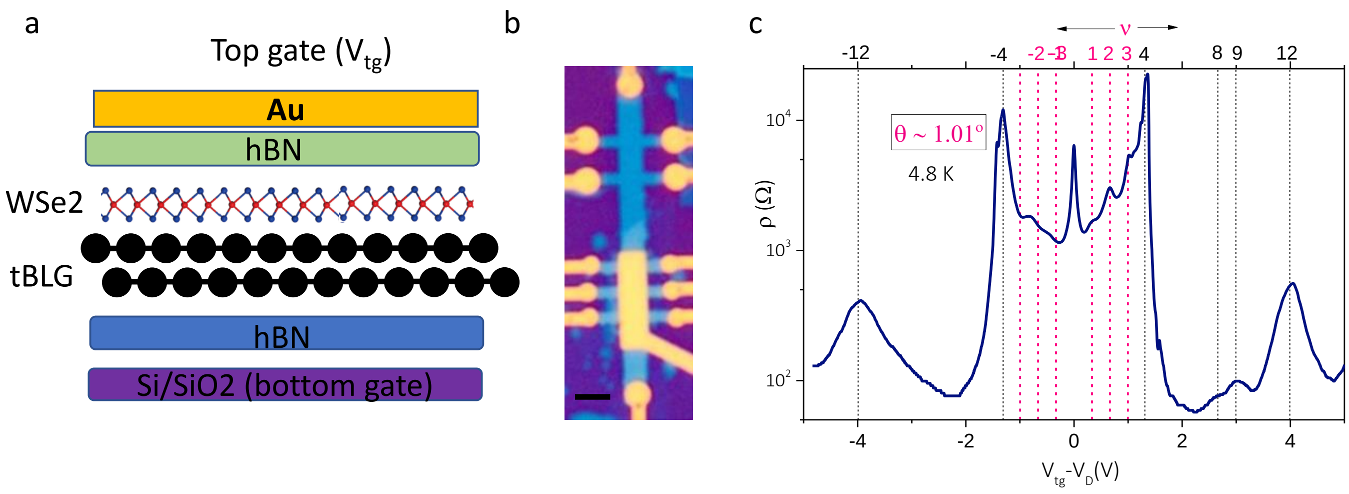

Supplementary Fig. 4: Device geometry and electric transport in device D1. (a) Schematic cross-sectional view of the device consisting of a multilayer WSe_2 sandwiched between the top hBN and the tBLG. (b) Optical micrograph of the device. The scale bar represents a length of 2 μm. (c) ρ as a function of gate voltage measured at 4.8 K.

B. Temperature dependence of resistance in device D2

Supplementary Fig. 5(a) shows the resistivity ρ measured in the 1.24° device as a function of n and T. We observe three resistive peaks near integer fillings on the e-side. The peaks near $\nu = 1, 2$ show a weak insulating behaviour upto $30 - 35$ K (Supplementary Fig. 5(c)), whereas the peak close to $\nu = 3$ show a metallic dependence throughout the temperature range. Similarly on the h-side, we observe a resistive peak between $\nu = -1$ and -2, which shows an insulating behaviour upto $40 - 50$ K. These peaks can not be explained in single particle band picture and are attributed to e-e correlations present in the system. In the following discussion, we denote the peaks near $\nu = 1, \pm 2$ as CI and the metallic state near $\nu = 3$ as CS. To further understand the origin of these CI/CS features, we focus on $\rho - T$ data between 0 and 3 electrons per superlattice in Supplementary Fig. 5 (b). We notice that the peak near $\nu = 1$ starts moving gradually and at $T = 30 - 35$ K, it moves to $\nu = 1$. Similar shift of resistive peak can be traced near $\nu = 3$, however the peak near $\nu = 2$ does not show any substantial change in its position. This observed effect has previously been reported for magic tBLG angle devices and is attributed to isospin Pomeranchuck effect arising due to small isospin stifness in these systems [6, 7], however the origin of such shifts in ρ peaks is still not very clear and requires further understanding.

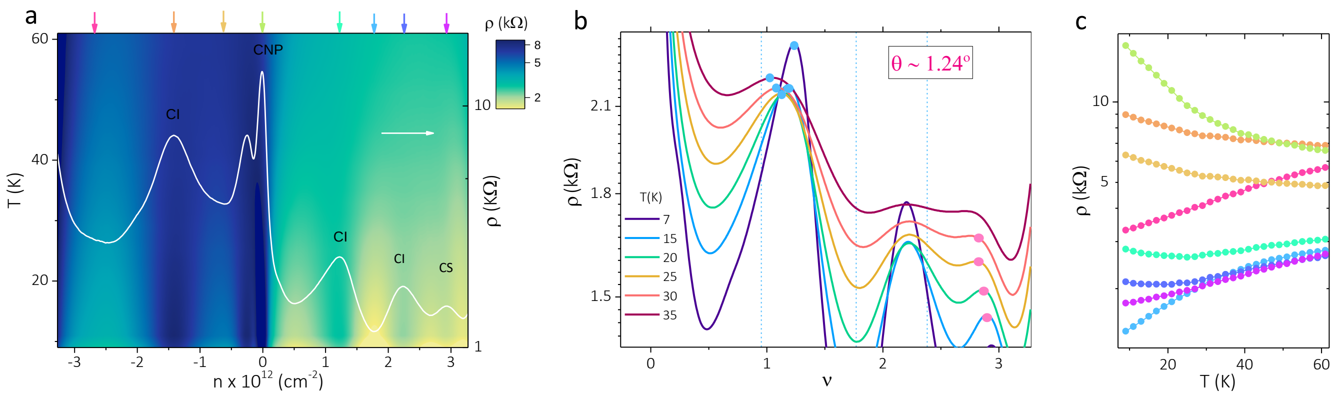

Supplementary Fig. 5: Temperature dependence of resistance for device D2(a) Temperature dependence of ρ in the 1.24° device, which exhibits multiple correlated resistive features close to integer fillings. The Right axis shows the R measured at 9 K. (b) ρ as a function of carrier density at a few selected temperatures. Light blue and pink circles indicate the shift in peak positions at $\nu = 1$ and 3. (c) $\rho(T)$ measured at different densities as marked by the arrows at the top in (a).

C. Resistance measured in different configurations for device D3 (1.6°)

We have measured the electrical resistance in multiple contact configurations to probe the twist angle inhomogeneity in our device. Optical micrograph of the device along with the contact configuration is shown in Supplementary Fig. 6(a), where the dashed lines show the two graphene layers and the metal top gate. Supplementary Fig. 6(b) shows a comparison of four terminal resistance measured at 3 K in three different configurations. Even though the magnitude of the resistance is slightly different in various configurations , the device exhibits a consistent moiré unit size in all configurations, as is evident by the good alignment of insulating states ($\nu = \pm 4$), thereby ensuring angle homogeneity. Further, we have measured the temperature dependence of resistance in the configuration given by, $I: 3-8, V: 2-9$. Supplementary Fig. 6(c),(d) show the resistance in the T linear regime. We find that R is metallic at $T \lesssim T_{\rm H}$, where $T_{\rm H} \sim 100-200$ K is a doping-dependent characteristic temperature. The results obtained in this configuration are consistent with the transport data shown in Fig. 1. Furthermore, it is to be noted that results shown in Supplementary Fig. 6(c),(d) are from a different thermal cycle as compared to Supplementary Fig. 6(b).

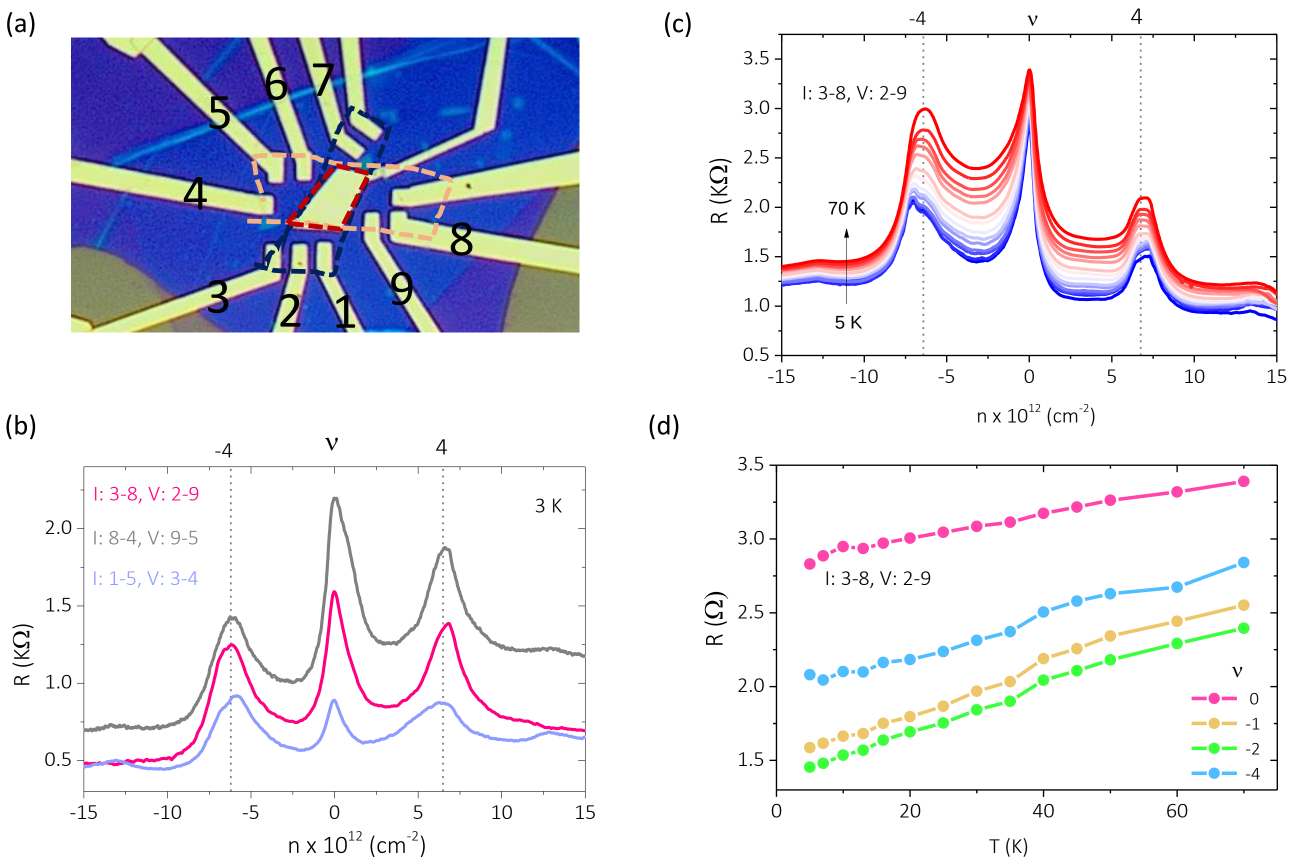

Supplementary Fig. 6: Electric transport measurements in different configurations in device D3. (a) Optical micrograph of the device D3 with the contact configuration. (b) Measured resistance (R) for three different configurations. Dashed lines show filling factor $\nu = \pm 4$. (c) R as a function of density at different T ranging from 5 K to 70 K in the configuration marked by, $I : 3-8, V : 2-9$. (d) T-dependence of R at different filling factors (ν) in the T-linear regime.

D. Electrical transport in different thermal cycles in device D3

Supplementary Fig. 7(a) shows the transport data from the 1.6° device in one of the thermal cycles (labelled as 'a'), where the resistance shows a shallow peak near $\nu \sim \pm 2$. We observe a clear electron-hole asymmetry in the R peaks, which is likely to emerge from the asymmetric band structure of tBLG. Similar shallow peaks in resistance reported in previous studies are attributed to van Hove singularities in the non-interacting density of states (DOS) which in-turn lead to large scattering cross-section for quasiparticles [8, 9]. However, we notice that the R peaks are suppressed by the application of a parallel magnetic field and finally vanishes at a field of 6(2) Tesla for $\nu \sim 2(-2)$ (Supplementary Fig. 7(b)). Furthermore, we have measured the T-dependence in a small range to probe the origin of these peaks (Supplementary Fig. 7(c)). Although the magnitude of the peak decreases at higher T ($7-8$ K), it is not possible to extract any quantitative information from the T dependence because of limited data. However, these features almost vanish after a thermal cycle to the sample (labelled by 'b') as shown in Supplementary Fig. 7(d). We wish to emphasise that the peaks in resistance near $\nu \sim \pm 2$ can not give rise to a 'peak' or 'trough' in thermopower, it must show a zero crossing at these filling, as observed in 1.24° device. Hence the origin of the excess thermopower cannot be connected to the appearance of these peaks.

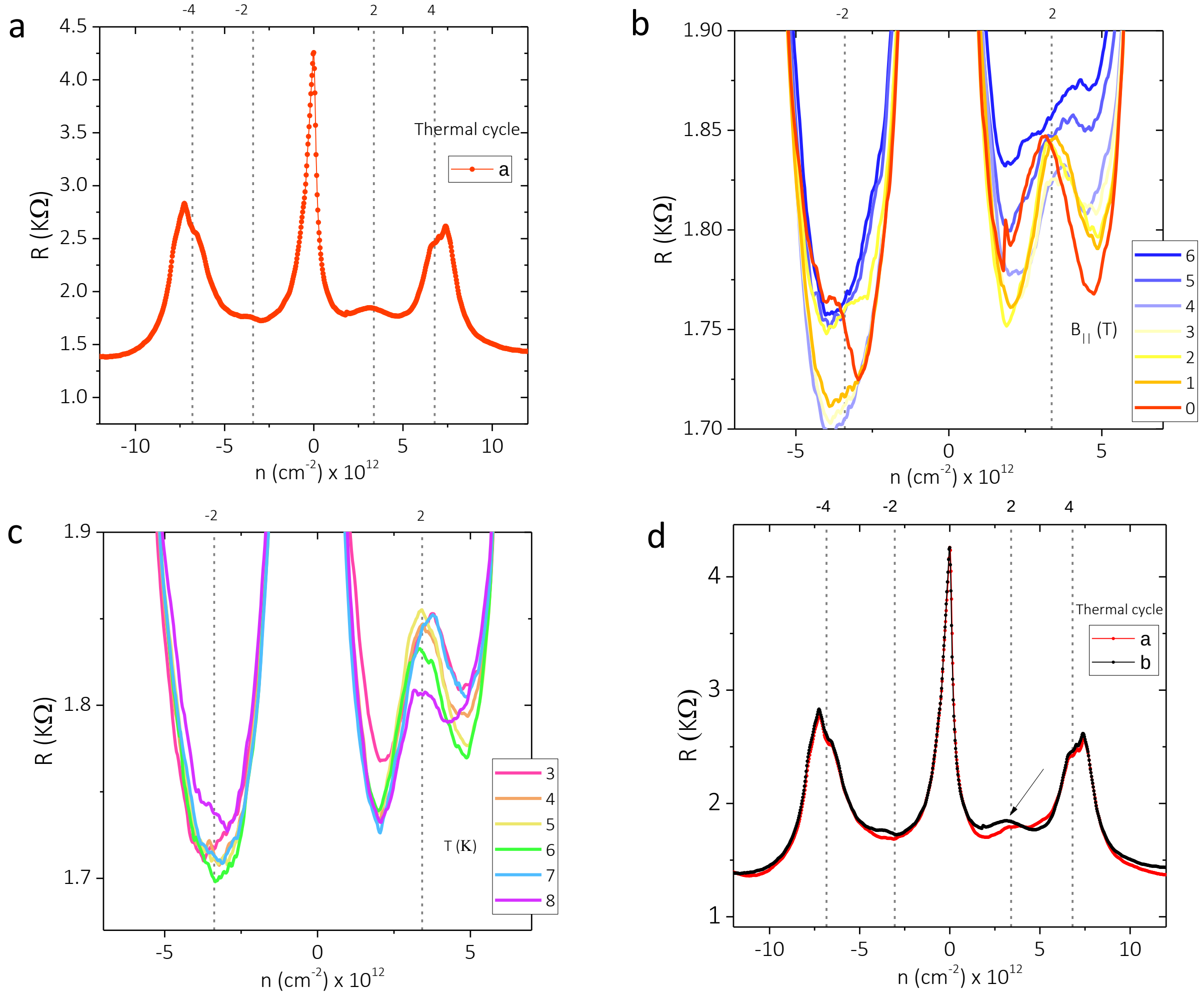

Supplementary Fig. 7: Electric transport in device D3 in different thermal cycles. (a) R as a function of of n for device D3 in thermal cycle number 'a' (b) Parallel Magnetic field dependence of R, zoomed-in to focus on R peak at $\nu = \pm 2$. (c) T dependence of resistance peak near $\nu = \pm 2$ (d) R plotted for two different thermal cycles.

E. Temperature dependence of resistance in device D3

Supplementary Fig. 8(a),(b) show ρ in the T-linear regime across a wide range of carrier density between the CNP and $\mp n_s$. In this T range, transport can be assumed to be restricted to the lowest electron-and hole- superlattice subbands. The dashed lines are linear fits to $\rho(T)$. A quantitative analysis of the fitted slope versus the density is shown in Fig. 4c. Furthermore, an insulating behaviour near $\pm n_s$ is observed in electrical transport as T is increased above $\approx$ 90 K. To study the thermally activated transport behaviour of insulating states, we plot the temperature dependence of resistance at $\nu = \pm 4$ in Supplementary Fig. 9(a),(b). An Arrhenius-like behaviour is clearly evident in this temperature range. From the slope in the Arrhenius plot, we estimate the activation gaps to be $\sim$ 160 and $\sim$ 240 K for the electron-side and hole-side respectively.

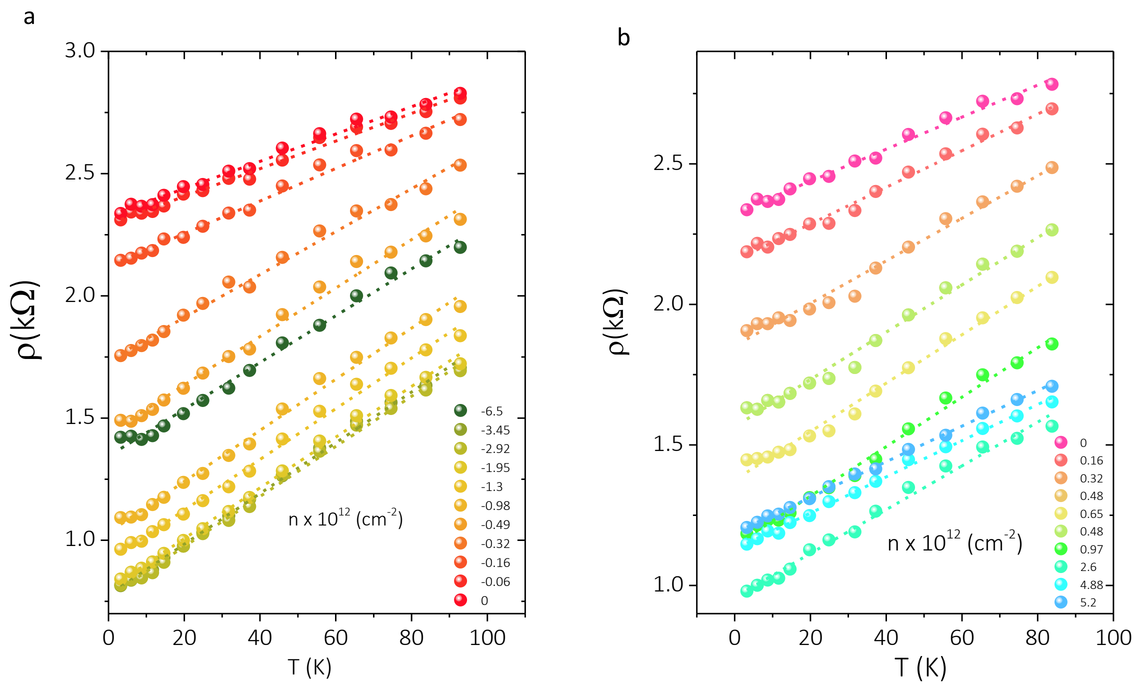

Supplementary Fig. 8: Temperature dependence of ρ at different n in device D3. (a),(b) ρ as a function of T for selected values of n in the T-linear regime. Dashed lines show linear fits to the data.

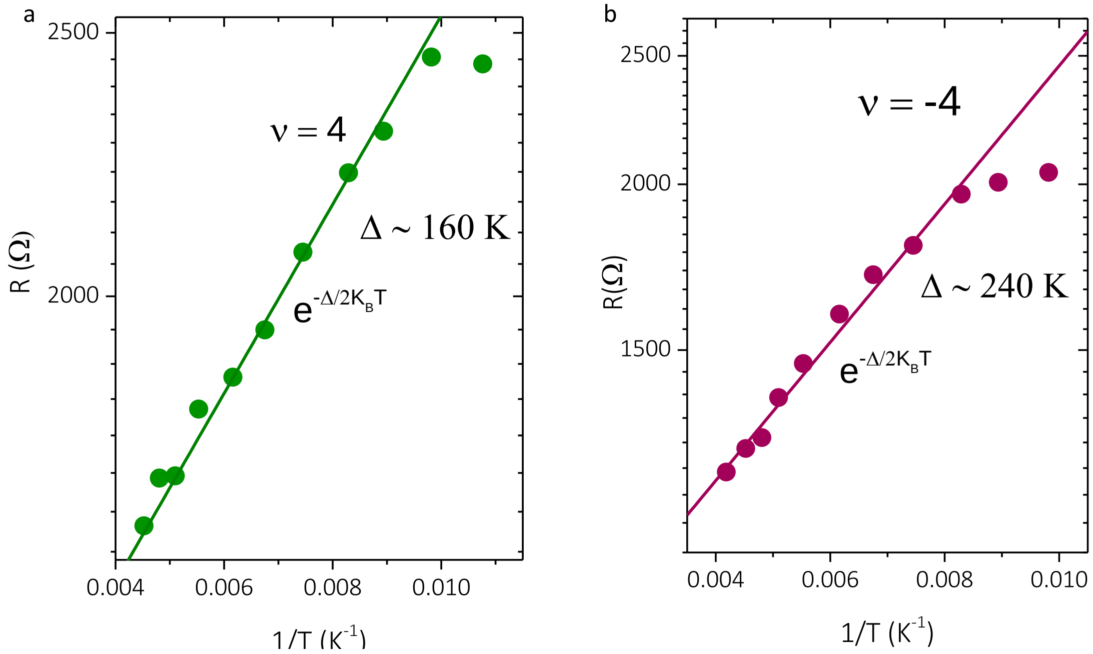

Supplementary Fig. 9: Temperature dependence at full filling of bands in device D3. (a),(b) R as a function of $1/T$ plotted for (a) $\nu = 4$, (b) $\nu = -4$. Solid lines show the Arrhenius-like fits to the data.

F. T-linear resistivity in D1 and D2 devices

Supplementary Fig. 10(a), (b) show the linear slope of resistivity versus density for 1.24° and 1.01° respectively. Notably, we have restricted our analysis of $d\rho/dT$ to $\nu = \pm 3$, since at higher densities the insulating states at $\nu = \pm 4$ start influencing $\rho(T)$. Additionally, shaded regions in the two graphs correspond to regions which show insulating behaviour at low T. We find that the D5 device exhibits higher values of $d\rho/dT$ as compared to D4, which is consistent with previous studies, where $d\rho/dT$ reaches a maximum value closer to magic angle [10, 11]. Notably, in both the devices $d\rho/dT$ attains a maximum value near $\nu = -3$ and shows a clear e-h asymmetry.

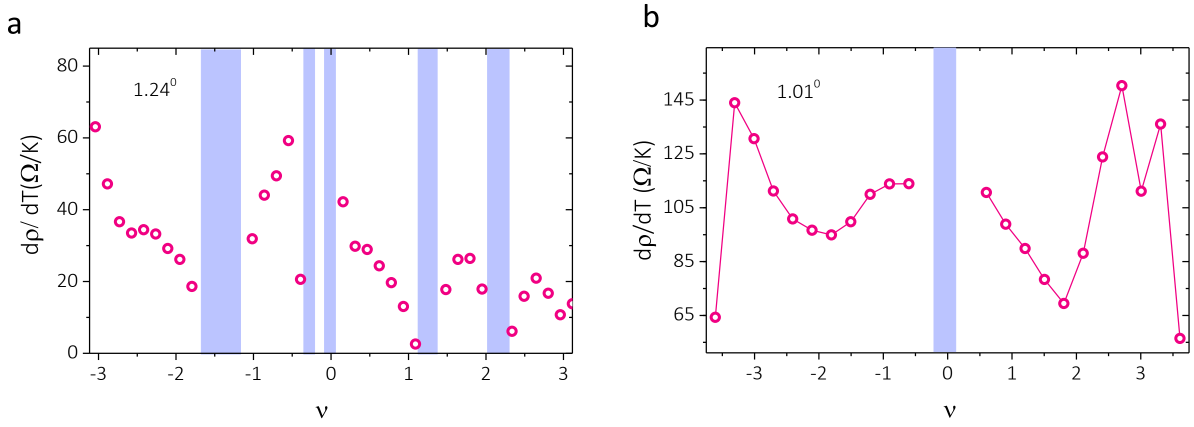

Supplementary Fig. 10: T-linear resistivity in devices D1 and D2. $d\rho/dT$ extracted in the T-linear regime for (a) 1.24° and (b) 1.01° devices . The shaded regions represents the insulating regions observed in the two devices.

Supplementary Note 5: Thermoelectric (TE) measurements in two different device geometries

We have carried out thermopower measurements in two different configurations depending on the device geometry, although the basic idea remains the same. For geometry C1, the measurements are schematically explained in Supplementary Fig. 11(a). Briefly, a sinusoidal current (I_ω) is allowed to flow between two contacts (e.g. 1 and 2) of the monolayer branch outside the top gated region, setting up a temperature gradient (ΔT) across the tBLG region. The resulting second-harmonic thermo-voltage ($V_{2\omega}$) is recorded between leads 3 and 4 as a function of doping and heating current.[4, 12]. The thermopower results presented in main text for device D3 are obtained in this configuration, additionally this geometry allows us to measure thermopower in different configurations which are shown in section V. Similary for H1 geometry, SLG region is used to create a (ΔT) across the tBLG channel, however in this case the voltage probes directly contact the tBLG channel as shown in Supplementary Fig. 11(b). We find that our thermopower results are consistent in two geometries and hence are intrinsic to tBLG. We wish to emphasize that the employed method of generating ΔT by heating one side of the device and measuring the thermal voltage across the channel has also been frequently used in 2D systems such as GaAs-AlGaAs[13, 14] and in graphene-based devices[15, 16]. It is to be noted that at ultra-low temperature, thermal conductivity of Si substrate is quite low to generate sufficient ΔT, and thus connected heater seems to be better way to achieve a reasonable ΔT.

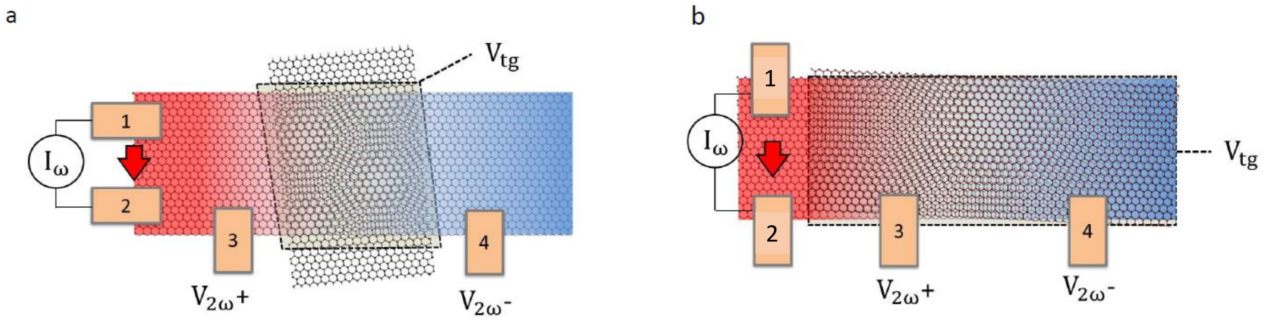

Supplementary Fig. 11: Device geometry for thermoelectric measurements. Measurement schematic for thermo-voltage $V_{2\omega}$ in (a) C1 and (b) H1 device geometries.

Supplementary Note 6: Additional data on thermoelectric transport in different devices

A. Thermoelectric transport in device D2

Supplementary Fig. 12(a) shows the density dependence of $V_{2\omega}$ measured for different heating currents. The linear response was ensured from $V_{2\omega} \propto I_\omega^2$ for the range of heating current used (Supplementary Fig. 12(b)). The evolution of $V_{2\omega}$ with n is explained in Fig. 2 (main text). In brief, the system shows a sequence of CI (near $\nu = 1$ and 2) and CS (near $\nu = 3$) states as the Fermi energy approaches integer fillings, and correspondingly $V_{2\omega}$ exhibits a cascade of sign reversals or thermovoltage oscillations. (Shaded light blue regions near $\nu = 1, 2$ and 3). We note that, the h-side does not show a clear sequence of thermopower oscillations at integer fillings, which further highlights the e-h asymmetry present in the system. In order to connect the resistive features and thermopower, we compare the scaled $V_{2\omega}$ with $\alpha = (1/R)dR/dn$ in Supplementary Fig. 12(b). Notably, while α qualitatively captures the sign reversals near integer fillings on the e-side, it fails to predict the excess thermopower near $\nu = 0.5$ (light pink shaded region), where $\alpha \approx 0$, which indicates a breakdown of the Mott formula as it is proportional to α. In addition, we observe a similar peak near $\nu = -1$ as well, which is also not captured by α, however the magnitude of excess thermopower is very less compared to the e-side.

In Supplementary Fig. 12(c) we make a qualitative comparison between the measured thermopower and the calculated values from the Mott formula (S_{Mott}), where we have used the calculted DOS for 1.24° to compute S_{Mott}. We find that S_{Mott} predicts much higher values of thermopower for $1 < |\nu| < 2$ as compared to the measured $V_{2\omega}$. The observed behaviour is likely to originate from the fact that the DOS used is calculated from single-particle tight-binding approach, where e-e correlations are not included and hence does not represent the actual DOS in the device. Furthermore, to see the effect of temperature on thermopower, we show the normalized $V_{2\omega}$ and the corresponding α for three different temperatures in Supplementary Fig. 12(d),(e) and (f). We find that the excess thermopower near $\nu = 0.5$ persists down to 280 mK and the qualitative behaviour of thermopower does not change as we go down in T.

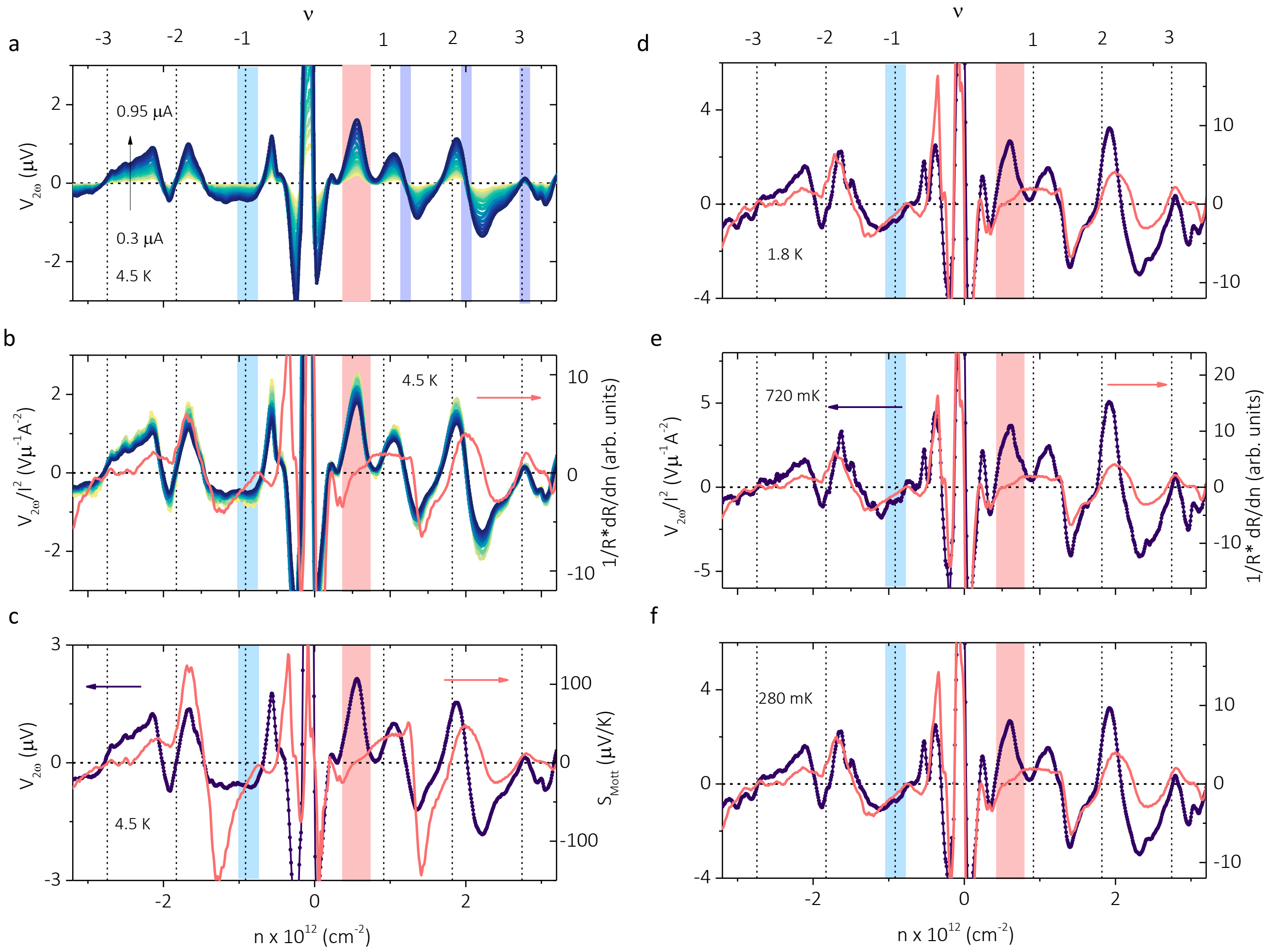

Supplementary Fig. 12: Thermoelectric transport in device D2 (a) $V_{2\omega}$ as a function of n for different heating currents $(0.4 - 0.9\ \mu\text{A})$ at 4.5 K. (b) $V_{2\omega}$ normalized with I_{ω}^{2} at 4.5 K. The right axis in (b) shows the numerically calculated $(1/R)\mathrm{d}R/\mathrm{d}n$. (c) Comparison between the measured $V_{2\omega}$ (Left axis) and that calculated (right axis) from the semiclassical Mott relation (Eq. 2 in main text). (d) (e) (f): Comparison of $V_{2\omega}$ (left axis) with corresponding $(1/R)\mathrm{d}R/\mathrm{d}n$ (right axis) at 1.8 K, 720 and 280 mK respectively. Dashed lines in all graphs show the integer fillings. Light orange (cyan) shaded region represents excess thermopower on e(h) side, whereas light blue regions in (a) represent the gapped/semimetallic phases.

Thermoelectric transport in device D2 at higher temperature

The thermoelelectric measurements shown in Supplementary Fig. 12 were performed in a wet dilution refrigerator, where the maximum accessible T was 4.5 K. In order to probe the behaviour of excess thermopower and CI/CS states at higher T, the device was measured in a different cryostat in another thermal cycle. We note that the device characteristics degraded in this cycle; however, most of the features were reproduced qualitatively. Supplementary Fig. 13 shows the thermopower data measured at five different temperatures, where $V_{2\omega}$ is normalized with respect to the CNP in order to focus on the excess therompower and the correlated CI/CS phases. We note that the excess thermopower near $\nu = 0.5$ (albeit very weak compared to the previous thermal cycle) starts vanishing around $10-12$ K. On the contrary, we find that the CI/CS phases persist upto $20 - 25$ K, pointing to a different physical mechanism responsible for the two features even though both originate from e-e interactions in the system. However, further understanding requires theoretical considerations and more experiments.

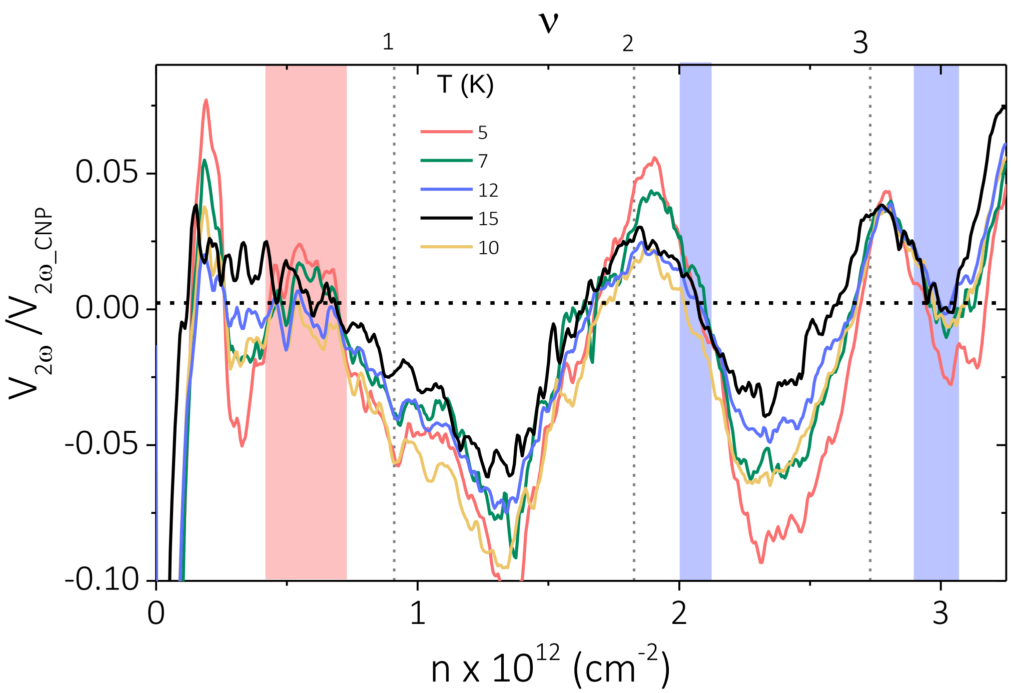

Supplementary Fig. 13: Thermoelectric transport in device D2 in second thermal cycle. $V_{2\omega}$ as a function of n for five different temperatures. The CI/CS states are marked by light blue shaded regions, whereas the light pink region highlights the excess thermopower peak.

B. Seebeck coefficient fitted with Mott formula in device D3

The fitting of the Mott formula (S_{Mott}) obtained using the calculated density of states (DOS) for three different twist angles with the measured thermopower for the 1.6° device is depicted in Supplementary Fig. 14. We observe that S_{Mott} from the DOS for 1.61° matches well with measured $V_{2\omega}$ both at the CNP and $\nu = \pm 4$ simultaneously, whereas if the twist angle is tuned away from 1.6°, we find a deviation in the Mott fit at $\nu = \pm 4$ as clearly shown for 1.69° and 1.41°. This further emphasises that the thermoelectric transport is extremely sensitive to the band structure and in turn any correlation effects, which can not be probed with conductance measurements alone.

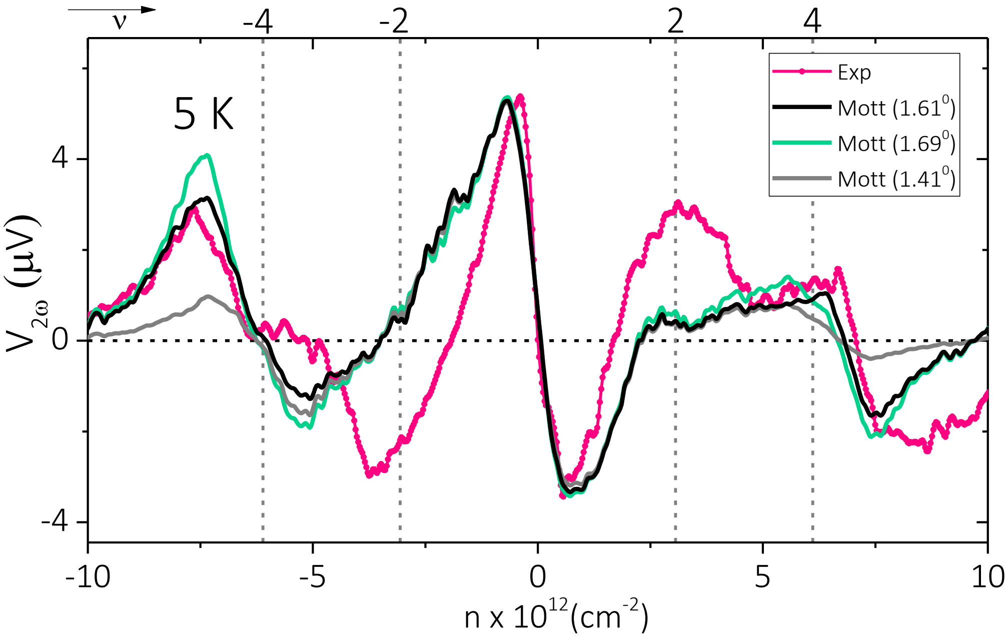

Supplementary Fig. 14: Comparison between measured thermopower and that calculated using Mott formula using different density of states in device D3. Doping dependence of the measured $V_{2\omega}$ compared with S_{Mott} calculated using DOS for three different twist angles 1.61°,1.69° and 1.41°.

Seebeck coefficient fitted with Mott formula at various temperatures in device D3

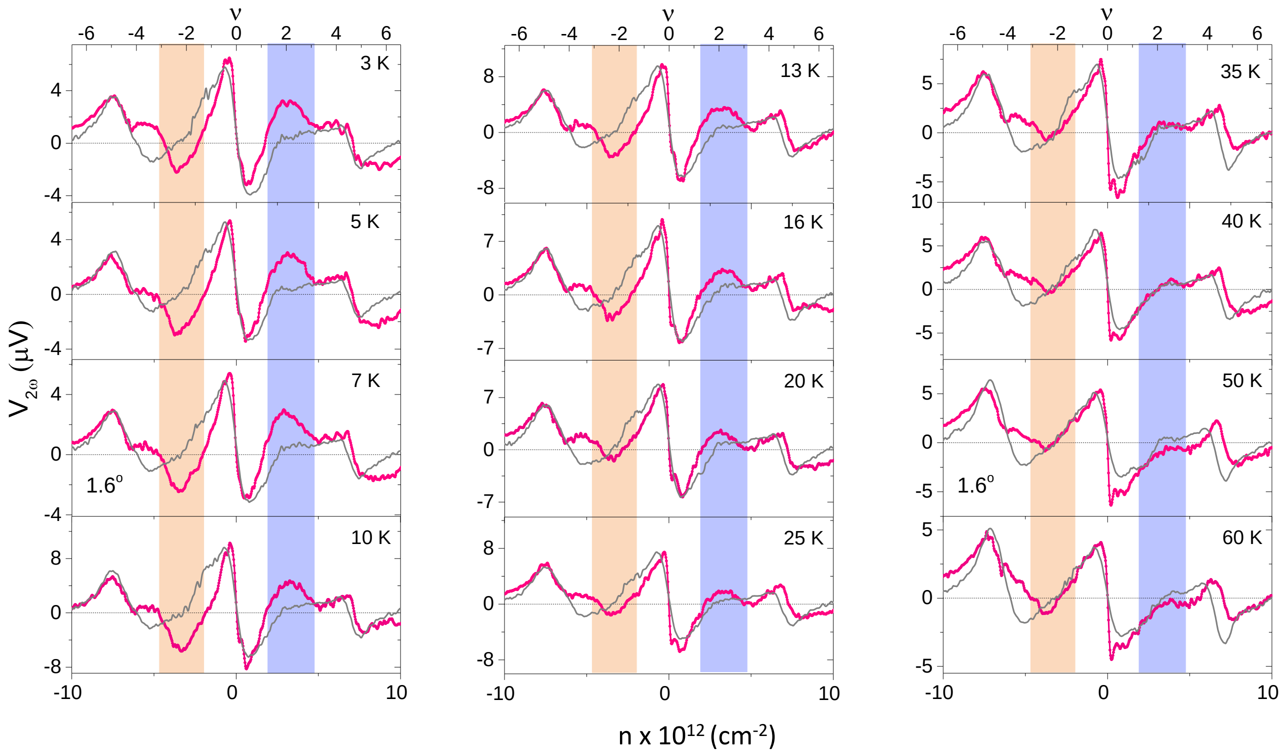

Supplementary Fig. 15: Comparison between measured thermopower and that calculated using Mott formula at different temperatures in device D3. Comparison between the measured $V_{2\omega}$ (pink lines) and that calculated (Grey line) from the semiclassical Mott relation at different temperatures.

C. ΔT calibration for device D3

The thermoelectric power (TEP) or Seebeck coefficient S is obtained from the ratio of thermoelectric voltage and the temperature gradient ($V_{2\omega}/\Delta T$) across the tBLG. In this work, we have used Mott formula to estimate the ΔT. This employed method of obtaining ΔT from Mott fitting is robust and frequently used in graphene-based devices[15, 17, 18]. Using ΔT as the single fitting parameter, we obtain excellent agreement between the measured $V_{2\omega}$ and that calculated using Mott formula near the CNP ($\nu \sim 0$), $\nu \sim \pm 4$, and also in the higher energy dispersive band ($\nu > \pm 4$) simultaneously (Fig. 3b and Supplementary Fig. 15). This suggests that the temperature gradient (ΔT) in the tBLG channel is independent of the doping and only depends on the heating current at the SLG region. We also note that the observed agreement between measured thermopower and Mott formula is expected in absence of correlation effects, which is the case near the CNP and in higher dispersive bands, thereby justifying our method of obtaining ΔT. We have fit the Mott formula to $V_{2\omega}$ at different values of T and obtained ΔT as shown in Supplementary Fig. 16(a). Additionally, as evident from the Supplementary Fig. 16(b), (c) and (d) the obtained Seebeck coefficient shows T-linear dependence in the dispersive band as expected from the T-linear dependence from Mott relation, further validating the employed method.

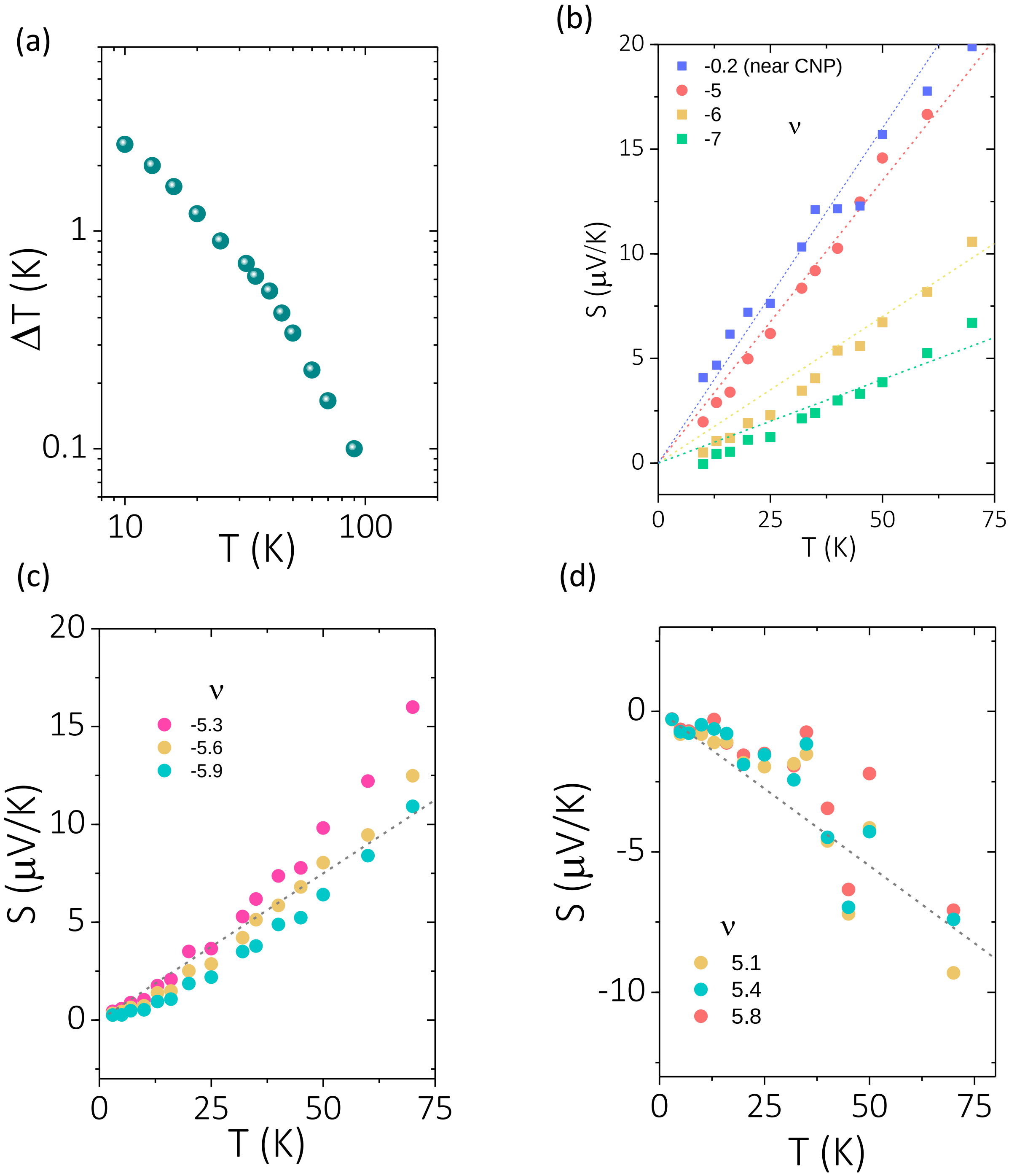

Supplementary Fig. 16: Estimation of ΔT in device D3. (a) The extracted values of ΔT obtained from Mott fit at various T. (b),(c) and (d) S as a function of T at CNP and at different fillings in higher dispersive bands. Dashed lines show the T-linear behaviour as a guide for eye.

To further justify our method of obtaining ΔT, we show thermopower data from a different twisted bilayer graphene sample ($\theta \sim 2°$), where we have used resistance thermometry to calculate the ΔT independently. We find excellent agreement between the measured $V_{2\omega}$ and that calculated using Mott formula in the entire density range as shown in Supplementary Fig. 17(a). In Supplementary Fig. 17(b), we plot ΔT obtained using the resistance thermometry and by fitting the Mott formula to the experimental data. Excellent agreement between the two justifies our method of

obtaining ΔT. We note that the resistance thermometry is not particularly effective at low temperature and hence we have used Mott formula to obtain the ΔT for device D3.

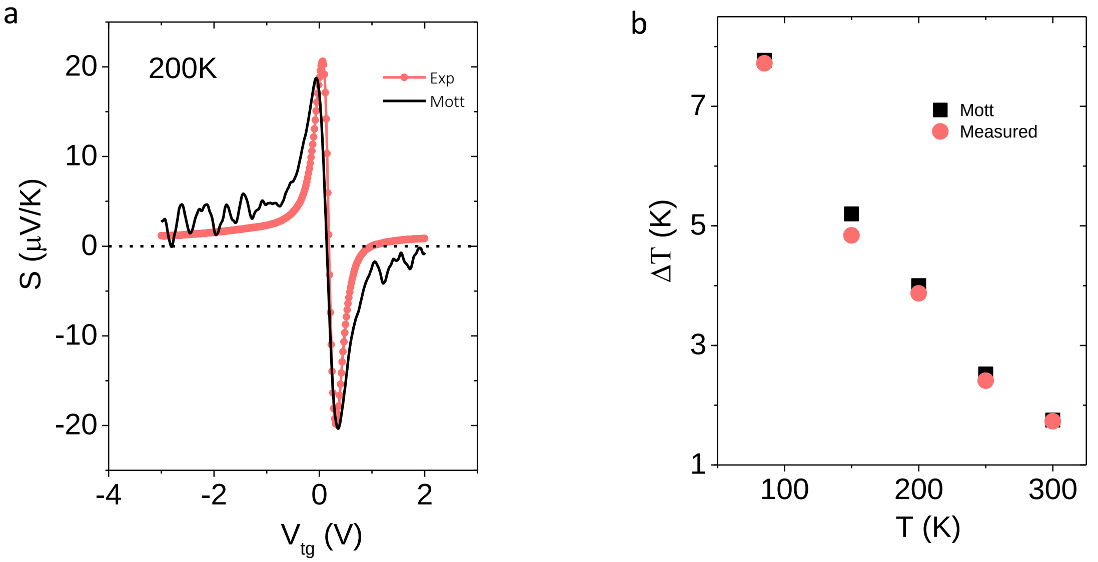

Supplementary Fig. 17: Estimation of ΔT using Mott formula and resistance thermometry in a twisted bilayer graphene sample with $\theta \sim 2°$. (a) The doping dependence of experimentally measured S (connected coloured circles) compared to S_{Mott} (black solid line) (b) Comparison of ΔT obtained from resistance thermometry and that extracted using Mott formula at various T.

D. Estimation of T_F for device D3 using tight-binding model

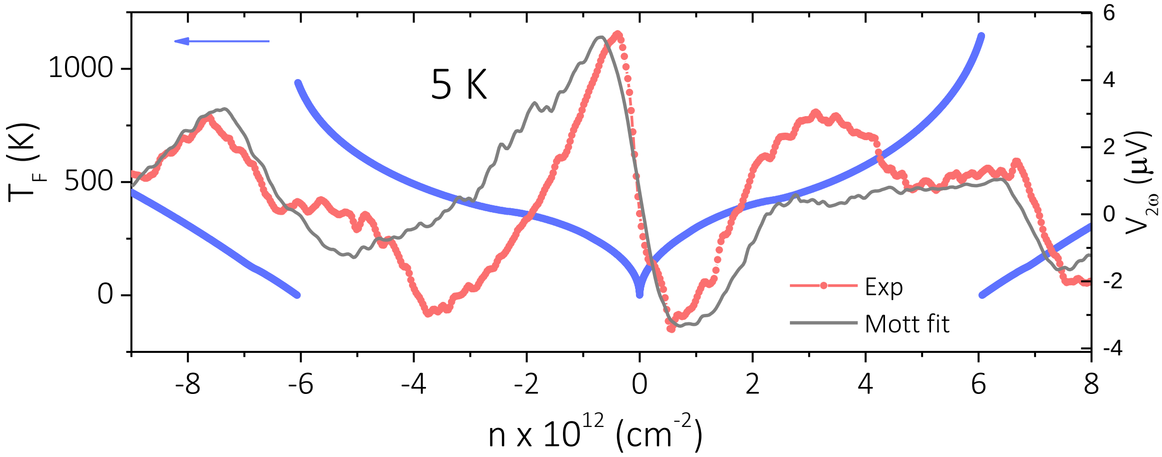

Supplementary Fig. 18: Estimation of Fermi Temperature in device D3. Doping dependence of T_F (left axis) calculated using tight-binding model plotted with the measured $V_{2\omega}$ and calculated thermopower using Mott formula (right axis). The Fermi temperature (T_F) is obtained from the band structure calculation by converting the energy scale to temperature. However, we have explicitly assumed that the effective number density of the conducting carriers is re-normalized to zero at $\nu = \pm 4$ due to the band gap. Consequently, T_F goes to zero at $\nu = \pm 4$, since the effective number density is zero. We perform the fitting at the local maxima/minima of $V_{2\omega}$ which is slightly away from the CNP ($n \approx 0.6 \times 10^{12}$ cm^{-2}), where we find $T_F \approx 240$ K. This satisfies the degenerate limit $T << T_F$ required for the validity of the Mott relation. Similarly, near the local maxima close to $|\nu| = 4$ ($n \approx 7.6 \times 10^{12}$ cm^{-2}), the value of $T_F \approx 250$ K and satisfies the degenerate limit.

E. Doping dependence of $S - S_{\mathrm{Mott}}$ for device D3

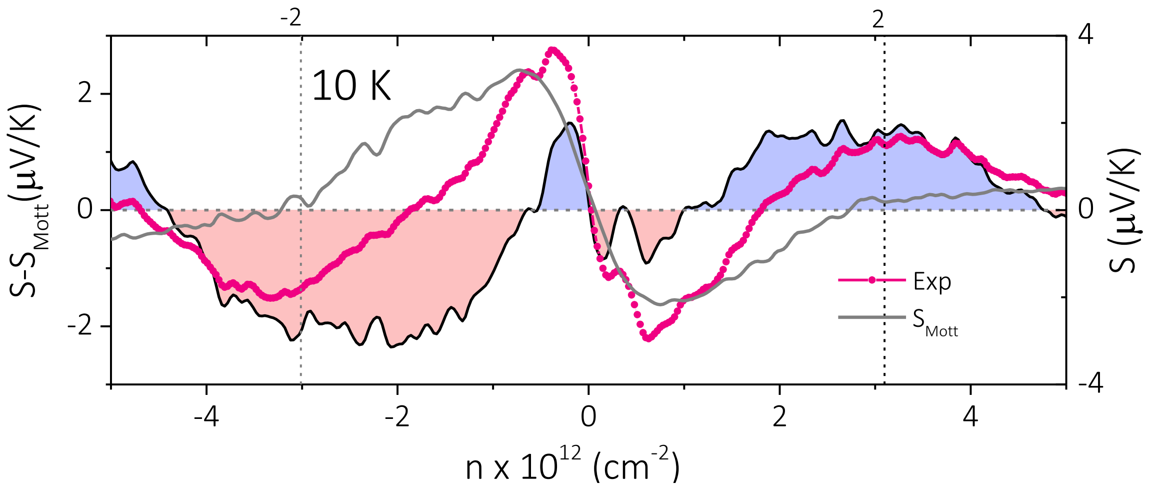

Supplementary Fig. 19: Excess thermopower in device D3. Doping dependence of measured $S - S_{\mathrm{Mott}}$ at 10 K, which attains maximum value near $\nu = 2$ on the e-side and between $\nu = -1$ and -2 on the h-side, reflecting the e-h asymmetry present in the system.

F. Thermoelectric measurements in magnetic field for device D3

Parallel field dependence

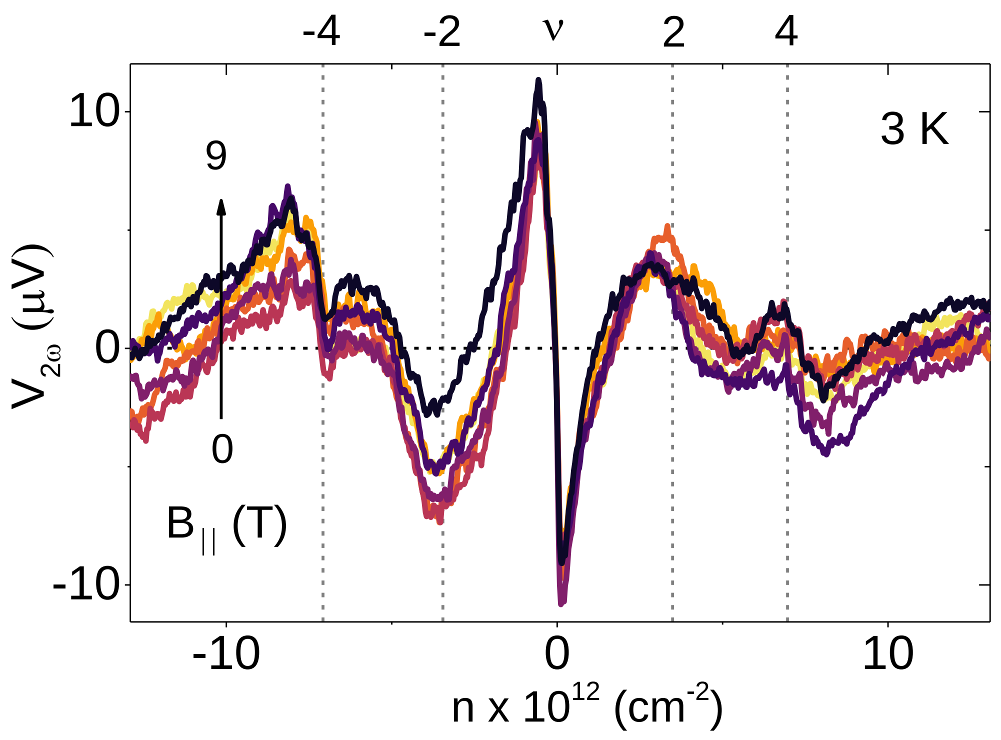

Supplementary Fig. 20: Thermopower measurements in parallel field in device D3. Thermopower $V_{2\omega}$ as a function of n for different magnetic fields applied parallel to plane of the tBLG measured at 3 K. No appreciable change in $V_{2\omega}$ is observed for magnetic fields upto 9 T.

Perpendicular field dependence

Supplementary Fig. 21 shows the low-field (perpendicular) normalized magneto-thermopower data. We find that the excess thermopower seems to diminish (more pronounced on h-doping) as we increase the magnetic field to 1 T and thus hints at the possibility of excess thermopower emerging due to orbital effects. However, it is to be noted that, we have not explored the thermopower behaviour in quantum hall regime as it is beyond the scope of the current manuscript and will be shown elsewhere.

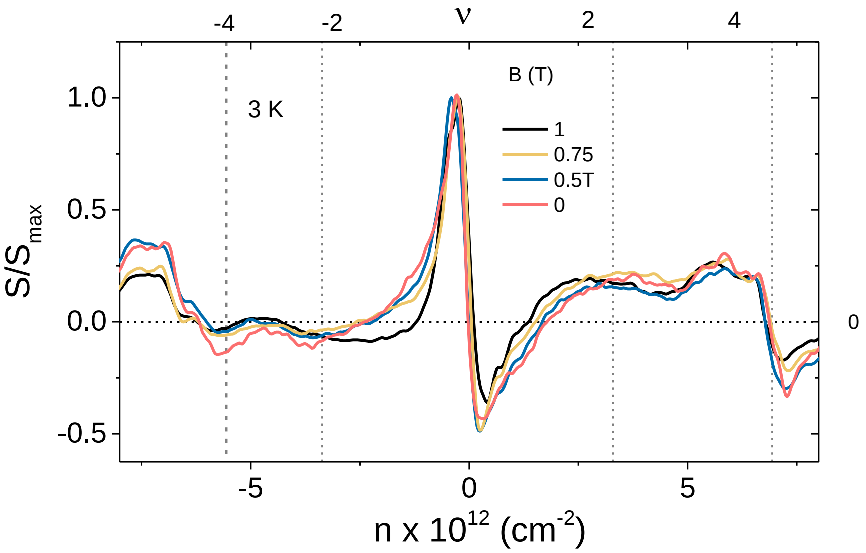

Supplementary Fig. 21: Thermopower measurements in perpendicular field in device D3. Normalized thermopower S/S_{max} as a function as a function of n for different magnetic fields applied perpendicular to plane of the tBLG measured at 3 K.

G. Thermoelectric measurements in different heating configurations in device D3

We have performed thermoelectric measurements in different heating configurations to verify the results described in Fig. 3. The optical micrograph of the device along with the contact configuration is shown in Supplementary Fig. 22(a). Briefly, a sinusoidal current is passed between two contacts to create a ΔT in the tBlG region and the resulting second harmonic voltage $V_{2\omega}$ is measured. Supplementary Fig. 22(b) shows the TE measured in three different configurations at 5 K. We note that apart from the sign reversal at the CNP and the vHS, two new extrema, consisting of a maximum close to $\nu = +2$ and minimum near $\nu = -2$ develop, which is very similar to the results described in main text (Fig. 3b). However, a slight asymmetry in different configurations is observed, which can arise from local inhomogeneity in the twist angle. Furthermore, we have performed temperature dependent TE measurements in the configuration given by, $I : 1 - 9, V : 3 - 4$. Supplementary Fig. 23 shows $V_{2\omega}$ for six different temperatures. We note that as T is increased above $35 - 40$ K, the excess thermopower near $\nu = \pm 2$ vanishes, indicating the T scale upto which correlation effects are present. Reproducibility of our thermopower results in different heating configurations ascertains the distinct nature of the ground state.

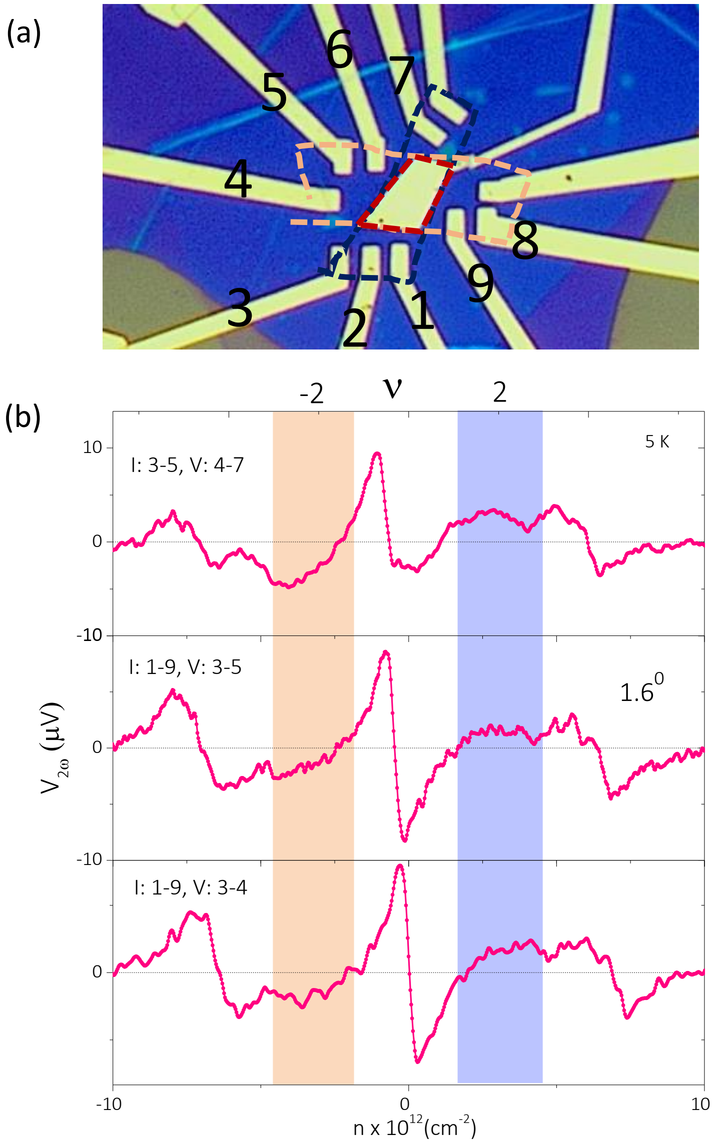

Supplementary Fig. 22: Thermopower measured in different configurations in device D3. (a) Optical micrograph of the device with the contact configuration. (b) Measured $V_{2\omega}$ for three different heating configurations.

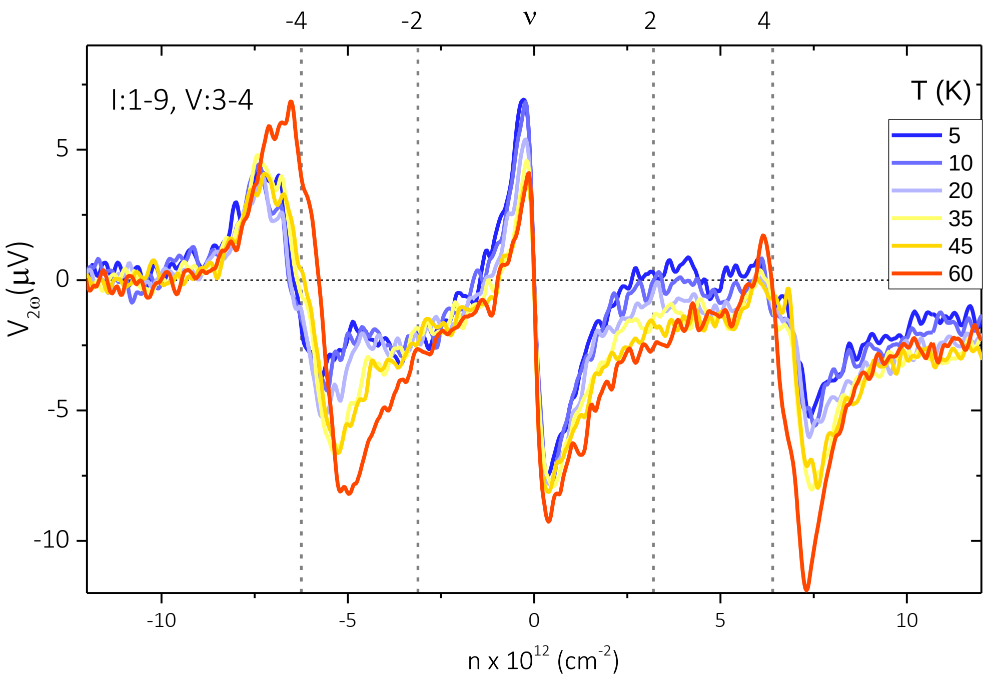

Supplementary Fig. 23: Temperature dependence of thermopower in device D3. Measured $V_{2\omega}$ for six representative T in the configuration $I : 1 - 9, V : 3 - 4$. Dashed lines show filling factors.

Supplementary Note 7: Planckian dissipation

In non-Fermi Liquid state (NFL), the incoherent scattering is often found to be bound by the Planckian dissipation scale $\tau^{-1} = C k_B T/\hbar$, where the dimensionless coefficient C is order of unity.

$$C = \frac{\hbar}{k_B} \frac{e^2 n_c}{m^*(0)} A \tag{1}$$

where n_c is the gate-induced carrier density and $m^*(0)$ is cyclotron mass at $T \to 0$ which is proportional to the density of states per Fermi pocket at the Fermi energy for two dimensional materials. We calculate the effective mass (m^*) as a function of n in the devices with twist angles 1.6°, 1.7° and 4° using the expression, $m^* = (h^2/2\pi)D(E)/N$, where N is the degeneracy. We have used N=8 (as evident from the Landau fan) and the density of states as calculated by tight binding method for 1.6°, 1.7° and the SLG DOS for 4°. Supplementary Fig. 24(a),(b) show the calculated m^* for the three devices. We note that our calculated values of m^* for 1.6° matches well with previously measured value for a device with similar twist angle [10]. The above expression allows us to calculate C and hence the scattering rate τ^{-1} over a range of number density as shown in Fig. 4d. We find that $C \sim O\ (1)$ for $\theta \approx 1.6°$ and 1.7° near $\nu = \pm 2$, where the deviation from Mott relation is maximum. In comparison, the value of C is two orders of magnitude smaller for $\theta \sim 4^{\circ}$.

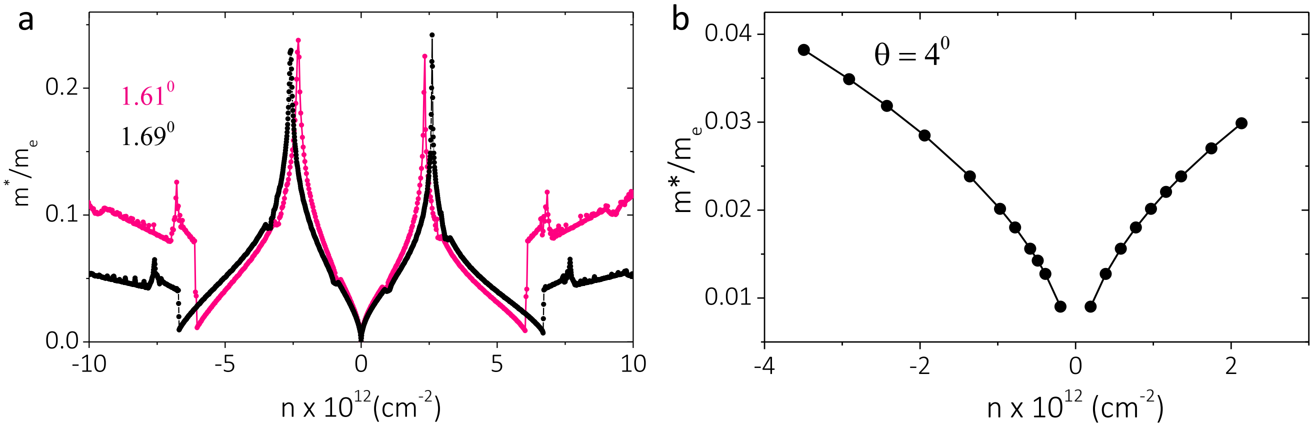

Supplementary Fig. 24: Cyclotron mass calculation. Numerically calculated cyclotron mass for (a) $\theta = 1.61°, 1.69°$ and (b) $\theta = 4°$
.

Supplementary Note 8: Theoretical calculations

A. Computational Details for the tight binding formalism

The electronic hamiltonian of the system is written as

$$\hat{\mathbf{H}} = -\sum_{i,j} t(\mathbf{R}_i - \mathbf{R}_j)c_i^\dagger c_j + \text{h.c.} = -\sum_{i,j} t_{ij} c_i^\dagger c_j + \text{h.c.} \tag{2}$$

where $\mathbf{R}_i$ denotes the real space position of the ith atom, and $c_i^\dagger$ and c_i are the creation and annihilation operators at $\mathbf{R}_i$. We approximate the transfer integrals t_{ij} using the Slater Koster formalism [19] assuming that the overlap of the p_z orbitals can be approximated as the linear combination of the $\sigma\sigma$ and the $\pi\pi$ overlaps. Taking the local curvature of the sheets into account the transfer integral can be written as [20]:

$$t_{ij} = t_{\pi\pi}[\hat{\mathbf{n}}_i - (\hat{\mathbf{n}}_i \cdot \hat{\mathbf{R}}_{ij})\hat{\mathbf{R}}_{ij}] \cdot [\hat{\mathbf{n}}_j - (\hat{\mathbf{n}}_j \cdot \hat{\mathbf{R}}_{ij})\hat{\mathbf{R}}_{ij}] + t_{\sigma\sigma}[\hat{\mathbf{n}}_i \cdot \hat{\mathbf{R}}_{ij}] \cdot [\hat{\mathbf{n}}_j . \hat{\mathbf{R}}_{ij}] \tag{3}$$

where $\hat{\mathbf{n}}_i$ is the unit normal at the ith site, and $\hat{\mathbf{R}}_{ij}$ is the unit vector joining the sites.
We can see that from Supplementary Fig. (25)that if there is no local curvature, and:

i. if i and j are on the same layer then the only non-zero contribution is from the $\pi\pi$ term

ii. if i and j are on different layers then $t_{ij} = t_{\sigma\sigma}\cos^2(\theta) + t_{\pi\pi}\sin^2(\theta)$ where θ is the angle that $\hat{\mathbf{n}}_i$ makes with $\hat{\mathbf{R}}_{ij}$

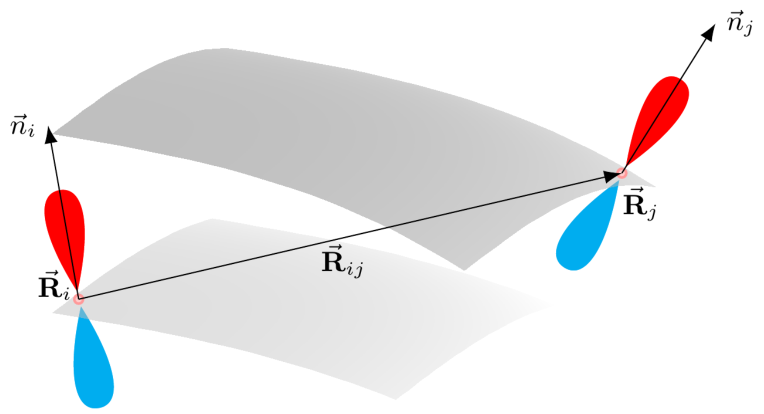

Supplementary Fig. 25: Local curvature of two sheets. Local normals at $\mathbf{R}_i$ and $\mathbf{R}_j$. Note that, $\hat{\mathbf{n}}_{i/j} = \frac{\vec{\mathbf{n}}_{i/j}}{|\vec{\mathbf{n}}_{i/j}|}$ and $\hat{\mathbf{R}}_{ij} = \frac{\vec{\mathbf{R}}_{ij}}{|\vec{\mathbf{R}}_j|}$

The terms $t_{\pi\pi}$ and $t_{\sigma\sigma}$ are taken as follows:

$$t_{\pi\pi} = t_\pi^0 \exp\Big(- \frac{|\vec{\mathbf{R}}_{ij}| - a_0}{\delta}\Big); \quad t_{\sigma\sigma} = t_\sigma^0 \exp\Big(- \frac{|\vec{\mathbf{R}}_{ij}| - d_0}{\delta}\Big) \tag{4}$$

On setting the parameter $a_0 = 1.42$ Å, the nearest neighbour distance between two Carbon atoms, it is reasonable to take $t_\pi^0 \approx -2.7$ eV, the nearest neighbour transfer energy in monolayer graphene. Similarly by taking $d_0 = 3.35$ Å, the interlayer distance in AA stacked bilayer, we fix $t_\sigma^0 \approx$ 0.48 eV. The attenuating factor, $\delta = 0.184\sqrt{3}a_0$ is chosen such that the strength of the next nearest neighbour is 0.1 times the nearest neighbour interaction in monolayer graphene.[21].
To compute the density of states we use an analog of the linear tetrahedron method for 2D systems, the linear triangulation method. A uniform (110×110) grid is taken in the Brillouin Zone (BZ) and a delaunay triangulation is performed on those points. The density of states and the number density are then estimated by integrating within each of the triangles.

B. Lattice model for the low-energy bands

To set up the dynamical mean field theory (DMFT) calculations involving the four low-energy bands near the CNP we assume an effective hexagonal lattice model for the moire lattice with two electronic orbitals and two spins $(\sigma = \pm 1/2)$ indexed by $\alpha = 1, \ldots, \mathcal{M}$ with $\mathcal{M} = 4$, such that there are four bands that can hold a maximum of eight electrons per triangular unit cell, i.e.

$$\mathcal{H} = -\sum_{ij,\alpha} t_{ij} c_{i\alpha}^\dagger c_{j\alpha} + \mathcal{H}_{int} \tag{5}$$

Here $c_{i\alpha}$ is the electron operator for i-th hexagonal lattice site. The hopping integrals are in general complex and can be chosen to fit [22–24] the energy dispersion from band-structure calculation, e.g. that shown in Fig. 3a of the main text. However, the two-orbital model is known [23, 25] to be insufficient to reproduce the topology of the Bloch wave functions while keeping all the low-energy symmetries of the continuum model [26]. Within the simplified DMFT approximation discussed below only the DOS of the low-energy bands enter and we take the DOS directly from our full tight-binding bandstructure calculation discussed in the main text. Hence the tight-binding parameterization for the low-energy effective lattice model does not explicitly appear in our DMFT calculations. We also neglect the asymmetry between the orbitals induced by the band structure. The interaction term $\mathcal{H}_{int} = U \sum_{\hexagon} (\sum_{i \in \hexagon, \alpha} n_{i\alpha}/3 - 4)^2$ is the cluster Hubbard term [22, 23] where the sum runs over all the hexagons in the moire lattice and $n_{i\alpha}$ is the electron number operator for α orbital/spin on the i-th site. The cluster term arises since the physical charges are concentrated around the AA regions of the moire lattice. When expanded the cluster Hubbard term leads to on-site as well as longer-range interactions with strength comparable to the on-site one [22]. The longer-range terms could be important

for various possible symmetry-breaking orders [22, 23, 27, 28] at low temperature near magic angles. Finding the phase diagram of the cluster Hubbard model for arbitrary filling is an interesting, but challenging theoretical problem [27, 28].

However, for the metallic state considered here within DMFT, the extended terms are mainly expected to lead to screening and renormalization of the on-site Hubbard U [29]. To this end, we take

$$\mathcal{H}_{int} = U \sum_{i,\alpha<\gamma} n_{i\alpha} n_{i\gamma}, \tag{6}$$

i.e. an $SU(4)$-symmetric on-site Hubbard interaction. The minimal model already captures the crucial effects of interaction near the low-energy vHS, as discussed below and in the main text. The strength of the on-site U could be estimated as $U = 1.857(e^2/4\pi\varepsilon\epsilon_0 L_{\mathrm{M}})$ [22, 30], where the moire lattice constant $L_{\mathrm{M}} = a_0/(2\sin(\theta/2)) = 8.89$ nm for graphene lattice spacing $a_0 = 0.246$ nm and twist angle $\theta = 1.6°$. Hence $U \simeq 15 - 75$ meV depending on the dielectric constant $\varepsilon \simeq 20-4$ or from screening due to the gates. Based on our estimated total band width of $W = 180$ meV for the four low-energy bands near CNP, we get a moderate interaction strength $U/W \simeq 0.1 - 0.4$. For our DMFT calculations, we take $U/W \simeq 0.2$. We find that the effect of this moderate interaction gets enhanced near the low-energy van Hove singularities (vHS).

C. Dynamical mean field theory

In the DMFT approximation [31], and assuming a homogeneous state with orbital and spin ($SU(4)$) symmetry, we reduce the model of Supplementary Eq.(5) to an effective single-site impurity problem with the imaginary-time action

$$\begin{aligned} S_{imp} = &-\int_0^\beta d\tau d\tau' \sum_\alpha \mathcal{G}^{-1}(\tau-\tau')\bar{c}_\alpha(\tau)c_\alpha(\tau') \\ &+ U\sum_{\alpha<\gamma}\int_0^\beta d\tau n_\alpha(\tau)n_\gamma(\tau) \end{aligned} \tag{7}$$

Here $\beta = 1/k_{\mathrm{B}}T$ and $(\bar{c}_\alpha, c_\alpha)$ are fermionic Grassmann variables with $n_\alpha = \bar{c}_\alpha c_\alpha$. The dynamical mean field $\mathcal{G}^{-1}(\mathrm{i}\omega_n) = \mathrm{i}\omega_n + \mu - \Delta(\mathrm{i}\omega_n)$ for the Matsubara frequency $\omega_n = (2n+1)k_{\mathrm{B}}T$, with n being an integer, is determined by the hybridization function $\Delta(\mathrm{i}\omega_n)$ which is self-consistently determined using the non-interacting lattice DOS as we discuss below. The chemical potential is fixed by the filling.

Since we need the real-frequency electronic Green's function $G(\omega) = G(\mathrm{i}\omega_n \to \omega + i0^+)$ to compute dc transport coefficients, e.g. the Seebeck coefficient, we use an approximate impurity solver, namely modified iterative perturbation theory (IPT) [31, 32] and its generalization for the multi-orbital case [33]. The latter has been benchmarked with numerically exact techniques for solving the single-impurity problem [33], and is expected to work quite well for moderate interaction strengths, like in our case. Within the IPT, we obtain the impurity self-energy as

$$\Sigma(\omega) = (\mathcal{M}-1)U\langle n\rangle + \frac{A(\mathcal{M}-1)\Sigma^{(2)}(\omega)}{1-B(\mathcal{M}-1)\Sigma^{(2)}(\omega)}, \tag{8}$$

and the impurity Green's function is obtained from the Dyson equation $G^{-1}(\omega) = \mathcal{G}^{-1}(\omega) - \Sigma(\omega)$. The first term in Supplementary Eq.(8) is the Hartree self energy and $\Sigma^{(2)}(\tau) = U^2\tilde{\mathcal{G}}^3(\tau)$ is the second-order self-energy obtained using the Hartree-corrected impurity Green's function $\tilde{\mathcal{G}}^{-1}(\omega) = \mathcal{G}^{-1}(\omega) - U\langle n\rangle$. The coefficients A and B are chosen to satisfy certain sum rules and the known high-frequency behaviour of the impurity Green's function [33] and are given by

$$A = \frac{\langle n\rangle(1-\langle n\rangle)}{\langle n_0\rangle(1-\langle n_0\rangle)} + \frac{(\mathcal{M}-2)(\langle nn\rangle - \langle n\rangle^2))}{\langle n_0\rangle(1-\langle n_0\rangle)} \tag{9}$$

$$B = \frac{(1-(\mathcal{M}-1)U\langle n\rangle) + \mu_0 - \mu}{(\mathcal{M}-1)U^2\langle n_0\rangle(1-\langle n_0\rangle)} \tag{10}$$

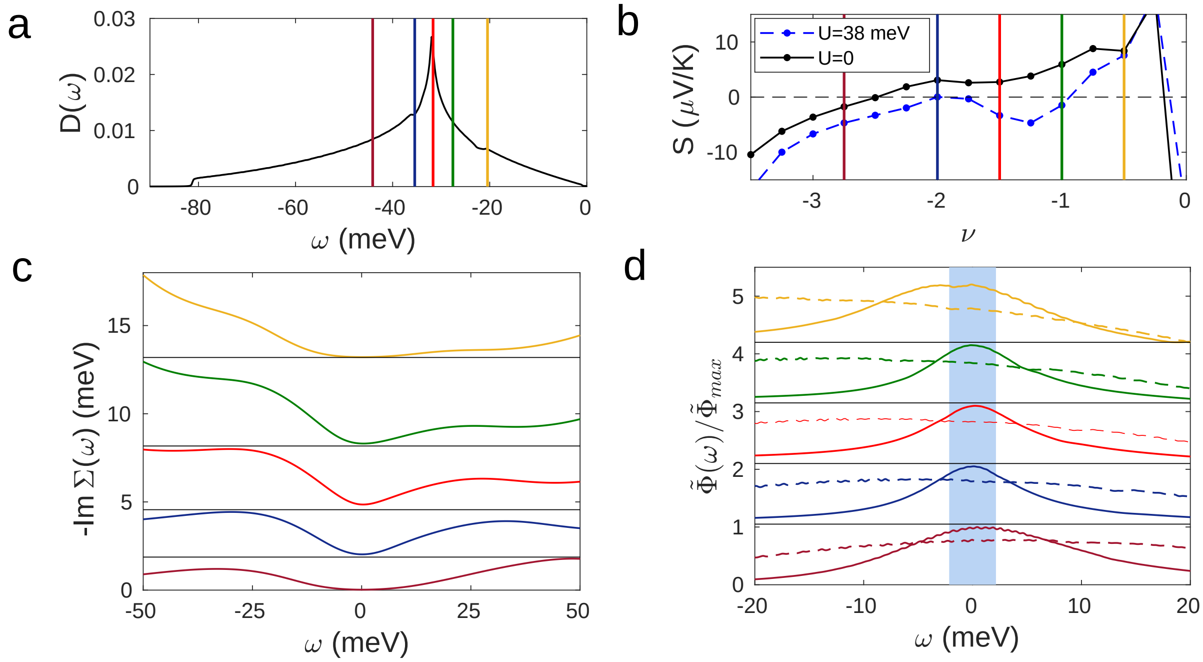

Supplementary Fig. 26: Van Hove singularity, self-energy and the sign of thermopower : (a) Non-interacting DOS from the tight binding bandstructure calculation for densities (or chemical potentials) below the CNP for $T = 14$ K. The vertical lines indicate five densities across the low-energy van Hove singularity (vHS). (b) The same densities or fillings (ν) are indicated by the vertical lines for the Seebeck coefficient (S) vs. ν plot at $T = 14K$ for the both non-interacting ($U = 0$, black line with filled circles) and the interacting ($U = 38$ meV, blue lines with filled circles) cases. The sign changes for the interacting and non-interacting cases happen at different fillings. (c) Imaginary part of electronic self energy at $T = 14$ K for the five fillings shown in (a) and (b) as indicated by the colors. For clarity, the self-energies at the subsequent fillings are vertically shifted with the baselines indicated by the solid horizontal lines. The electronic self-energy is enhanced near the vHS with marked low-energy particle-hole asymmetry. The Fermi liquid-like behavior, $-\mathrm{Im}\Sigma(\omega) \sim \omega^2$, at low energies persist over a narrower energy window near the vHS indicating a more correlated metallic state with a lower coherence temperature scale. (d) Effective transport DOS $\tilde{\Phi}(\omega)$ (solid lines) for the same five fillings as in (a), (b) and (c), indicated by the same colors. Again, $\tilde{\Phi}(\omega)$ for the five subsequent fillings are vertically shifted for clarity. The large self-energy effects near the vHS strongly modify the effective transport DOS for the interacting case compared to the non-interacting $\tilde{\Phi}(\omega)$ (dashed lines). The shaded region has a width $\sim k_{\mathrm{B}}T$ determined by $-T(\partial n_{\mathrm{F}}(\omega)/\partial\omega)$ that controls the integral [Supplementary Eq.(13)] appearing in Supplementary Eq.(12) for the Seebeck coefficient. The interacting $\tilde{\Phi}(\omega)$ becomes sharply peaked near the vHS. The sign of S depends on whether $\tilde{\Phi}(\omega)$ increases or decreases with ω near $\omega = 0$ (see text for a discussion).

Here $\langle n\rangle = -(1/\pi)\int_{-\infty}^{\infty} d\omega n_{\mathrm{F}}(\omega)\mathrm{Im}G(\omega) = (\nu+4)/8$ is the occupancy of a single orbital. The density-density correlation function $\langle nn\rangle \approx -\int_{-\infty}^{\infty} d\omega n_{\mathrm{F}}(\omega)\mathrm{Im}(\Sigma G)/(\pi U(\mathcal{M}-1))$ with $n_{\mathrm{F}}(\omega) = 1/(e^{\beta\omega}+1)$, the Fermi function. Following ref.34, we fix the 'pseudo' chemical potential μ_0 from $\langle n_0\rangle = -(1/\pi)\int_{-\infty}^{\infty} d\omega n_{\mathrm{F}}(\omega)\mathrm{Im}\tilde{\mathcal{G}}(\omega) = \langle n\rangle$ using the Hartree corrected Green function $\tilde{\mathcal{G}}^{-1}(\omega) = \omega + \mu_0 - \Delta(\omega) - U\langle n\rangle$.

Once the self-energy [Supplementary Eq.(8)] is obtained, the DMFT self-consistency condition is used to compute the local lattice Green's function

$$G(\omega) = \frac{1}{2\mathcal{M}}\int_{-\infty}^{\infty} d\epsilon \frac{D(\epsilon)}{\omega+\mu-\epsilon-\Sigma(\omega)}, \tag{11}$$

assumed to be the same as the impurity Green's function for a single orbital and spin species. Here $D(\epsilon)$ is the total tight-binding DOS per triangular moire unit cell for the four lowest bands. The self-consistency loop is closed by obtaining the new hybridization function $\Delta(\omega) = \omega + \mu - \epsilon - \Sigma(\omega) - G^{-1}(\omega)$. The loop is iterated till we reach convergence.

We fix the interaction strength $U = 38$ meV and perform the calculations to obtain electronic Green's function and self-energy for fillings $-3 < \nu < 3$ and temperatures 5K $\lesssim T \lesssim 100$ K. The Seebeck coefficient [35] S is given by,

$$S = -\frac{k_{\rm B}}{e}\frac{A_1}{A_0} \tag{12}$$

where

$$A_m = \int_{-\infty}^{\infty} d\omega d\epsilon \rho^2(\epsilon,\omega)\Phi(\epsilon)\left(-T\frac{\partial n_{\rm F}(\omega)}{\partial\omega}\right)(\beta\omega)^m \tag{13}$$

with $m = 1, 2$. $\rho(\epsilon,\omega) = -(1/\pi)\text{Im}(1/(\omega+\mu-\epsilon-\Sigma(\omega)))$ is obtained from DMFT self-energy and $\Phi(\epsilon) = (1/\mathcal{A})\sum_{n=1,\mathbf{k}}^{4}(\partial\epsilon_{n\mathbf{k}}/\partial k_x)^2\delta(\epsilon-\epsilon_{n\mathbf{k}})$ is the transport DOS that is obtained from tight-binding energy dispersion $\epsilon_{n\mathbf{k}}$ for the four bands near CNP. Here $\mathcal{A}$ is the area of the sample. To analyze the sign of the Seebeck coefficient, we define an effective transport DOS, $\tilde{\Phi}(\omega) = \int_{-\infty}^{\infty} d\epsilon\rho^2(\epsilon,\omega)\Phi(\epsilon)$. In the non-interacting case ($U = 0$), we take $\rho(\epsilon,\omega) \sim \eta/((\omega+\mu-\epsilon)^2+\eta^2)$ with a small phenomenological broadening $\eta \simeq 0.001W$ mimicking the effect of impurity scattering. In this case, $\rho^2(\epsilon,\omega)$ is sharply peaked around $\omega = \epsilon - \mu$ and $\tilde{\Phi}(\epsilon,\omega)$ is effectively determined by the tight-binding transport DOS $\Phi(\epsilon)$. As a result, at low temperatures A_1, A_0 and hence S is determined by the behavior of $\Phi(\omega+\mu)$ over an energy window $\sim k_{\rm B}T$ around the chemical potential ($\omega = 0$). Hence the sign of A_1 or the Seebeck coefficient is negative (positive) depending on whether $\Phi(\omega+\mu)$ increases (decreases) with ω, and $S \approx 0$ when $\omega = 0$ is at the peak of $\Phi(\omega+\mu)$. As we show in Supplementary Figs .26, for the interacting case treated within DMFT, due to the large and strongly temperature-dependent self-energy effects near the vHSs, $\tilde{\Phi}(\omega)$, unlike $\Phi(\omega+\mu)$, becomes very sharply peaked around the chemical potential and varies strongly over energy window $\sim k_{\rm B}T$. These lead to the non-standard sign and the violation of SMR over a range $-2 \lesssim \nu \lesssim -1$ as shown in Fig. 4(e) and Supplementary Fig. 27(a).

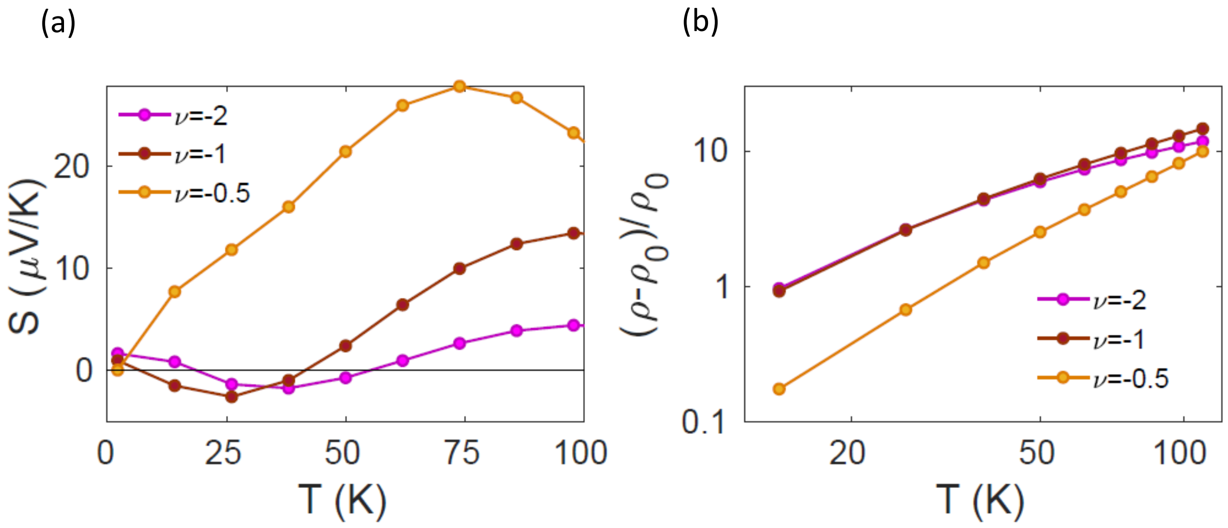

Supplementary Fig. 27: Seebeck coefficient calculated using DMFT. (a) Computed S from DMFT as a function of temperature for fillings $\nu = -2, -1$ and -0.5 . The Seebeck coefficient changes from positive to negative sign over an intermediate temperature range. (b) Normalized resistivity ρ computed from DMFT as a function of T for fillings $\nu = -2, -1$ and -0.5, where ρ_0 is the residual resistivity. The dashed line shows T-linear dependence.

As shown in Supplementary Fig. 26, we also find strong particle-hole asymmetry in the self-energy and DOS even at low-energies near $\omega = 0$ (chemical potential). The particle-hole asymmetry results from the particle-hole asymmetry

of the non-interacting DOS at intermediate energies away from the vHSs, as evident in Fig.3a. The asymmetry is further enhanced by the interaction. This asymmetry also influences the sign of the thermopower near the vHSs at low and intermediate temperatures. We also obtain a linear-T resistivity (Supplementary Fig. 27(b)) over an extended temperature range. This is, of course, a known outcome of DMFT for correlated systems [36, 37]. However, to obtain the detailed density dependence and the magnitude of resistivity one needs to consider the electron-phonon scattering and the effects of long-range impurities relevant at such low-densities near CNP [38, 39]. The thermopower and the violation of SMR are not expected to be influenced significantly by electron-phonon and impurity scattering [40, 41].

D. Violation of Mott relation in DMFT

In Supplementary Fig. 28(d), we show the deviation $\Delta S = S - S_{\rm Mott}$ from the Mott value $S_{\rm Mott}$ (Supplementary Fig.28(b)) obtained from resistivity ρ (Supplementary Fig. 28(a)) computed in DMFT using the Mott formula of Eq.1 of the main text. The computed ΔS is large in the intermediate temperatures ($< U/k_{\rm B}$), indicating strong violation of Mott law in DMFT, in contrast to that at low temperature. However, a comparison of this violation of the Mott law in DMFT to the experimentally observed violation of SMR is somewhat misleading, since the former mainly arises from the strong filling dependence of the DMFT resistivity, which exhibits additional broad peaks near $|\nu| = 2$, due to large elctron-electron (el-el) scattering near the van Hove singularity (vHS). Such additional peaks are absent in the experimentally measured resistivity (Fig. 1, main text), though seen during thermal cycles (Supplementary Fig. 7). Due to these additional resistivity peaks, the computed $S_{\rm Mott}$ in DMFT is large at intermediate temperatures and has additional sign changes (Supplementary Fig. 28(b)) compared to the actual thermopower S (Supplementary Fig. 28(c)), leading to large ΔS i.e. strong violation of Mott law (Supplementary Fig. 28(d)).

We would also like to emphasize that our DMFT calculations only capture intrinsic el-el contributions to resistivity and thermopower, and impurity scatterings, which leads to the residual resistivity ρ_0, is only treated in an approximate manner in terms of a filling independent impurity broadening in the DMFT Green's functions. Hence our calculations cannot capture the large residual resistance seen in experiment and its filling dependence, presumably arising from the details of the impurity scattering and its possible intricate interplay with el-el scattering at low-temperature. These effects are beyond the scope of our current theoretical calculations. However, as is well known [40], scatterings from static impurities alone , even if large, cannot give rise to violation of the Mott law. The excess thermopower in the DMFT calculations could be quantified better by comparing with the non-interacting results at $U = 0$ as shown in Supplementary Figs. 29(a),(b). Here, we can clearly deduce the excess thermopower and its opposite sign appearing close to $|\nu| = 2$ due to the enhanced el-el interaction near the vHSs.

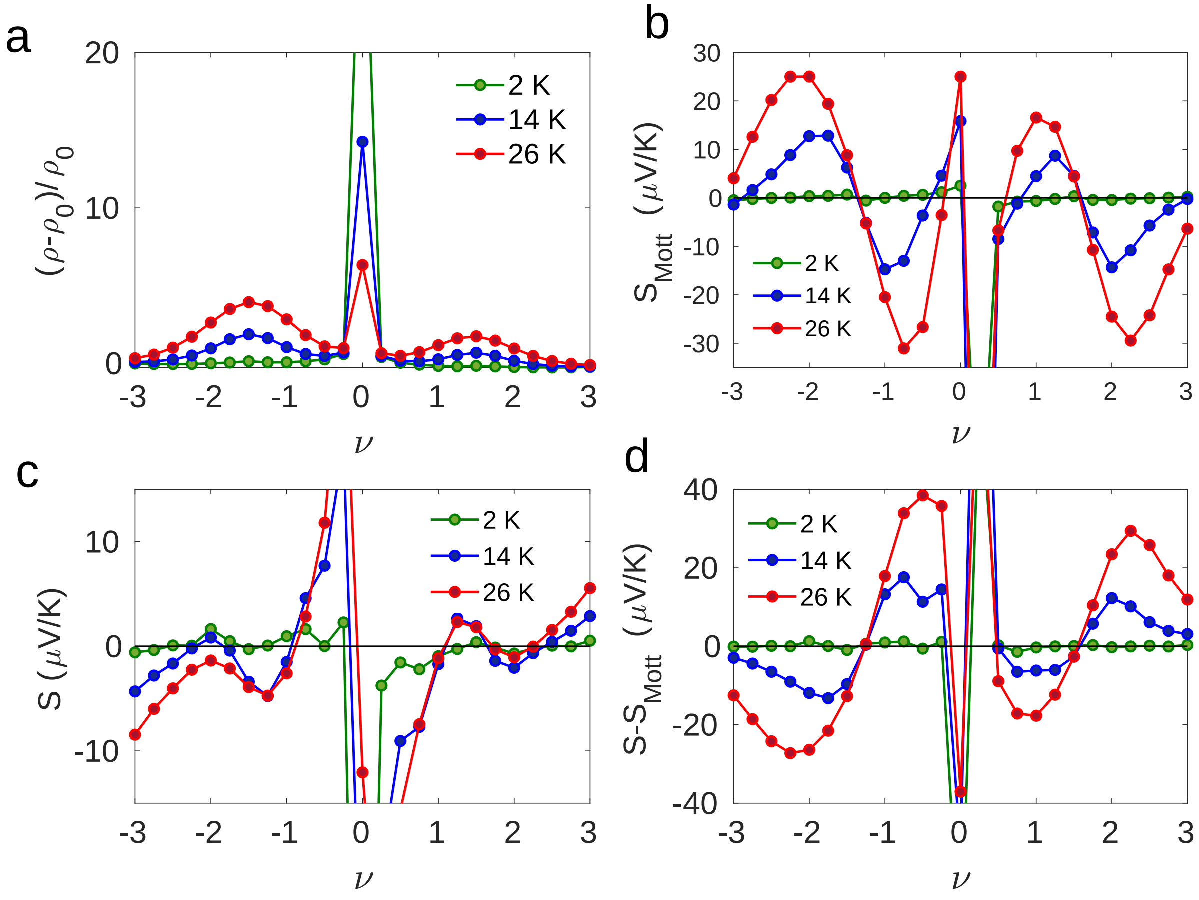

Supplementary Fig. 28: Violation of Mott relation in DMFT.(a) Normalized resistivity $(\rho - \rho_0)/\rho_0$ relative to the residual resistivity ρ_0 as a function of ν for $T = 2$,14, 26 K. (b) $S_{\mathrm{Mott}}(\nu)$ computed in DMFT using Mott law. (c) Thermopower S computed from DMFT. (d) The deviation $\Delta S = S - S_{\mathrm{Mott}}$ from Mott value in DMFT.

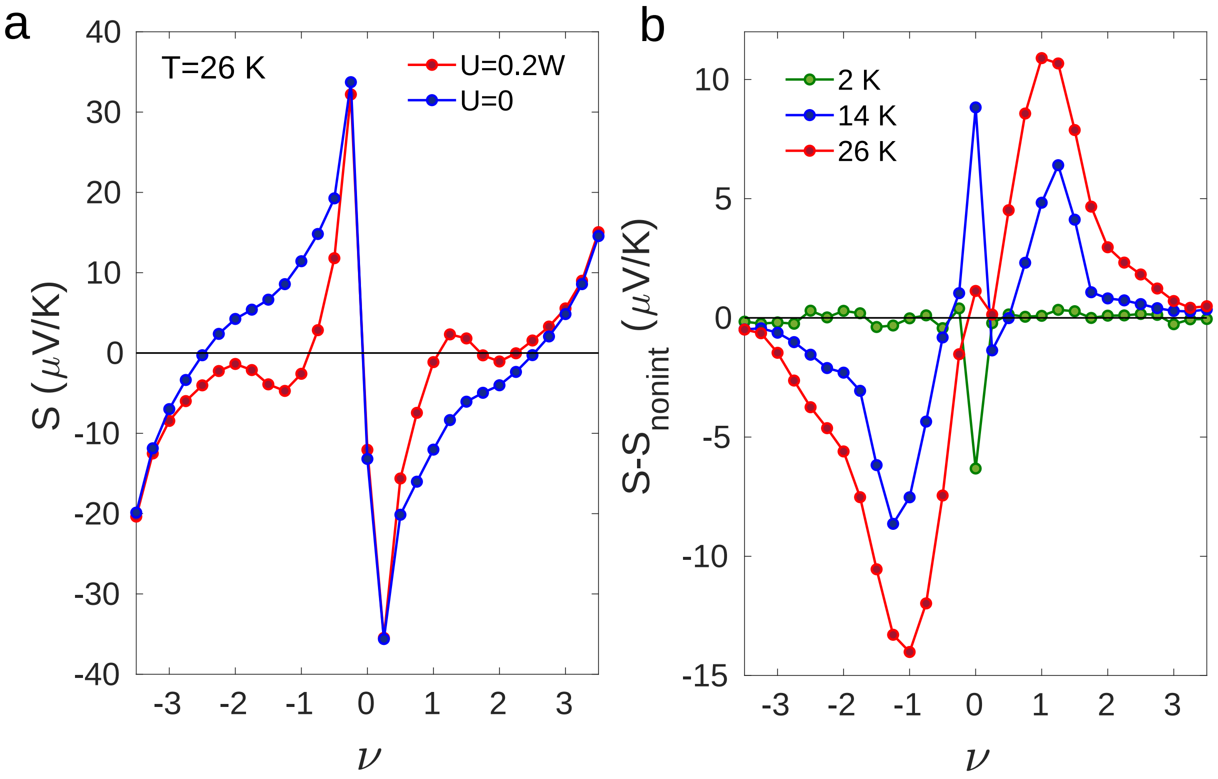

Supplementary Fig. 29: Thermopower calculated for interacting and non-interacting cases. (a) Comparison of thermopower for interacting ($U = 0.2W$) and non-interacting ($U = 0$) cases at $T = 26$ K. (b) The excess thermpower $S - S_{nonint}$ due to el-el interaction is large for $|\nu| \simeq 1 - 2$ near the vHSs.

E. Comparison between excess thermopower and entropy in DMFT

The connection between thermopower and thermodynamic entropy (S_{ent}) is typically understood in terms of so-called Kelvin formula [42, 43], $S = -(1/|e|)(\partial S_{ent}/\partial n)$, where n is the number density of the charge carrier. The Kelvin formula can be obtained for non-interacting electrons from the SMR (Eq.1, main text) by assuming that the only dependence of the resistance R on energy E is due to density of states, and the velocity and scattering time are energy independent. Hence the Kelvin formula is only an approximation. However, the formula works quite well for several strongly-correlated systems [42, 44], and the formula is found to be exact in certain models of non-Fermi liquid metals [43, 45] that are dual to charged black holes [46]. We do not find the Kelvin formula to describe our results for DMFT thermpower S well, presumably due to strong energy dependence of velocity due to the vHSs. However, as shown in Supplementary Fig. 30, we find the excess entropy due to el-el interactio, over the non-interacting ($U = 0$) entropy, to correlate well with the excess thermopower (Supplementary Fig. 29) as a function of filling and temperature. We compute the entropy (per hexagonal moire lattice site, spin and orbital) in DMFT using the Maxwell relation $(\partial S_{ent}/\partial n)_T = -(\partial \mu/\partial T)_n$, where $\mu(T)$ is obtained in DMFT by fixing the filling. We obtain the $S_{ent}(\nu, T)$ by integrating over $(\partial S_{ent}/\partial n)_T$ from $\nu = -4(4)$ to $\nu < 0(> 0)$, assuming $S_{ent}(|\nu| = 4, T) = 0$ i.e. when the low-energy band is completely empty or full. This is justified in our theoretical model which does not consider the higher bands.

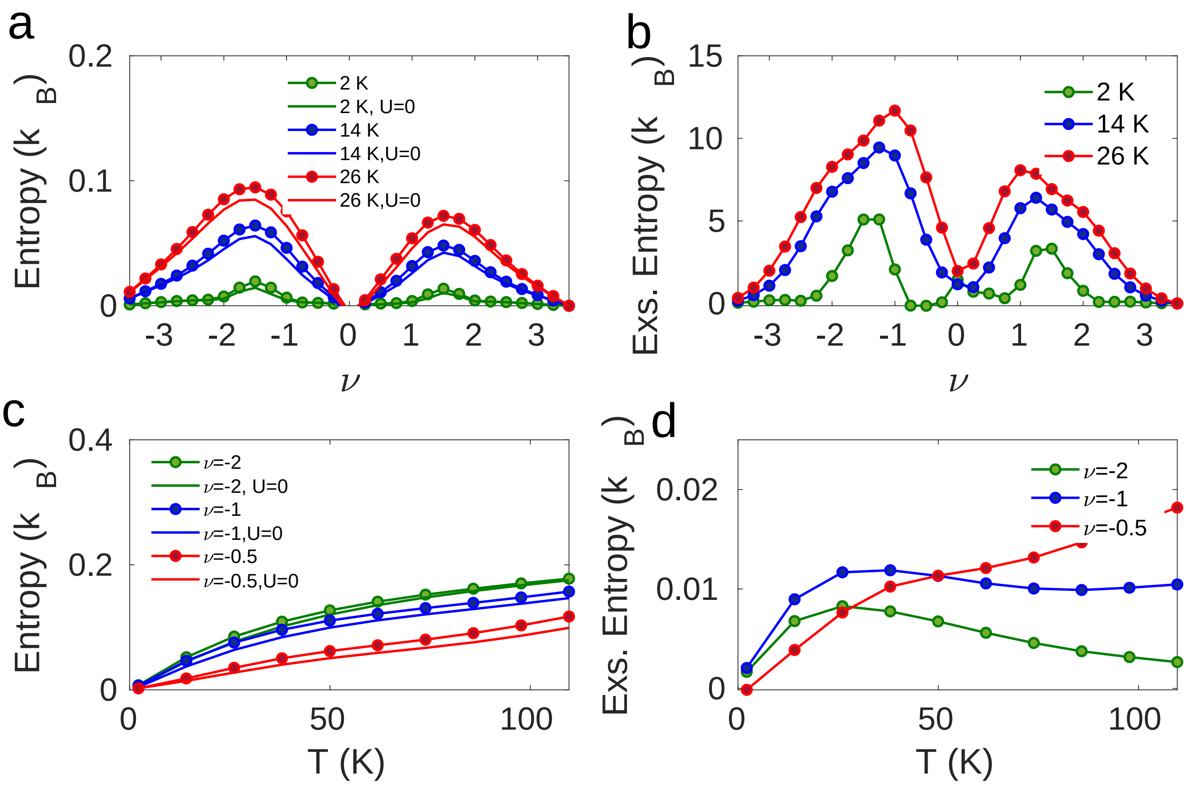

Supplementary Fig. 30: Comparison between excess thermopower and entropy in DMFT. (a) Comparison of entropy S_{ent} as a function of filling ν for $T = 2$, 14 and 26 K for interacting ($U = 0.2W$) and non-interacting ($U = 0$) cases. (b) The excess entropy $S_{ent}(U = 0.2W) - S_{ent}(U = 0)$ (in units of $10^{-3}k_{\mathrm{B}}$) as a function of ν. (c) Comparison of S_{ent} for interacting and non-interacting cases as a functions of temperature for $\nu = -2, -1, -0.5$ and (d) corresponding excess entropies as function of temperature.

B.G., P.S.M. and M.G. contributed equally to this work.

SUPPLEMENTARY REFERENCES

* gbhaskar@iisc.ac.in
† phanis@iisc.ac.in
‡ manjarigarg@iisc.ac.in
§ arindam@iisc.ac.in

[1] P. S. Mahapatra, B. Ghawri, K. Watanabe, T. Taniguchi, S. Mukerjee, and A. Ghosh, "Mis-orientation controlled cross-plane thermoelectricity in twisted bilayer graphene," (2019), arXiv:1910.02614 [cond-mat.mes-hall].

[2] R. W. Havener, H. Zhuang, L. Brown, R. G. Hennig, and J. Park, Nano Lett. **12**, 3162 (2012).

[3] K. Kim, S. Coh, L. Z. Tan, W. Regan, J. M. Yuk, E. Chatterjee, M. Crommie, M. L. Cohen, S. G. Louie, and A. Zettl, Phys. Rev. Lett. **108**, 246103 (2012).

[4] P. S. Mahapatra, K. Sarkar, H. R. Krishnamurthy, S. Mukerjee, and A. Ghosh, Nano lett. **17**, 6822 (2017).

[5] Y. Cao, J. Luo, V. Fatemi, S. Fang, J. Sanchez-Yamagishi, K. Watanabe, T. Taniguchi, E. Kaxiras, and P. Jarillo-Herrero, Phys. Rev. Lett. **117**, 116804 (2016).

[6] Y. Saito, J. Ge, K. Watanabe, T. Taniguchi, E. Berg, and A. F. Young, arXiv preprint arXiv:2008.10830 (2020).

[7] A. Rozen, J. M. Park, U. Zondiner, Y. Cao, D. Rodan-Legrain, T. Taniguchi, K. Watanabe, Y. Oreg, A. Stern, E. Berg, *et al.*, arXiv preprint arXiv:2009.01836 (2020).

[8] Y. Kim, P. Herlinger, P. Moon, M. Koshino, T. Taniguchi, K. Watanabe, and J. H. Smet, Nano letters **16**, 5053 (2016).

[9] T.-F. Chung, Y. Xu, and Y. P. Chen, Physical Review B **98**, 035425 (2018).

[10] H. Polshyn, M. Yankowitz, S. Chen, Y. Zhang, K. Watanabe, T. Taniguchi, C. R. Dean, and A. F. Young, Nature Physics **15**, 1011 (2019).

[11] Y. Cao, D. Chowdhury, D. Rodan-Legrain, O. Rubies-Bigorda, K. Watanabe, T. Taniguchi, T. Senthil, and P. Jarillo-Herrero, Physical review letters **124**, 076801 (2020).

[12] Y. M. Zuev, W. Chang, and P. Kim, Phys. Rev. Lett. **102**, 096807 (2009).

[13] R. Scheibner, H. Buhmann, D. Reuter, M. Kiselev, and L. Molenkamp, Phys. Rev. Lett. **95**, 176602 (2005).

[14] S. Goswami, C. Siegert, M. Baenninger, M. Pepper, I. Farrer, D. A. Ritchie, and A. Ghosh, Phys. Rev. Lett. **103**, 026602 (2009).

[15] A. Jayaraman, K. Hsieh, B. Ghawri, P. S. Mahapatra, K. Watanabe, T. Taniguchi, and A. Ghosh, Nano Letters **21**, 1221 (2021).

[16] J. Waissman, L. E. Anderson, A. V. Talanov, Z. Yan, Y. J. Shin, D. H. Najafabadi, M. Rezaee, X. Feng, D. G. Nocera, T. Taniguchi, *et al.*, Nature nanotechnology , 1 (2021).

[17] J. F. Sierra, I. Neumann, J. Cuppens, B. Raes, M. V. Costache, and S. O. Valenzuela, Nature nanotechnology **13**, 107 (2018).

[18] I. Vera-Marun, J. Van Den Berg, F. Dejene, and B. Van Wees, Nature communications **7**, 1 (2016).

[19] J. C. Slater and G. F. Koster, Physical Review **94**, 1498 (1954).

[20] S. Choi, J. Deslippe, R. B. Capaz, and S. G. Louie, Nano Letters **13**, 54 (2013).

[21] P. Moon and M. Koshino, Physical Review B **85**, 195458 (2012).

[22] M. Koshino, N. F. Q. Yuan, T. Koretsune, M. Ochi, K. Kuroki, and L. Fu, Phys. Rev. X **8**, 031087 (2018).

[23] H. C. Po, L. Zou, A. Vishwanath, and T. Senthil, Physical Review X **8** (2018), 10.1103/physrevx.8.031089.

[24] J. Kang and O. Vafek, Physical Review X **8** (2018), 10.1103/physrevx.8.031088.

[25] H. C. Po, L. Zou, T. Senthil, and A. Vishwanath, Physical Review B **99** (2019), 10.1103/physrevb.99.195455.

[26] R. Bistritzer and A. H. MacDonald, Proceedings of the National Academy of Sciences **108**, 12233–12237 (2011).

[27] X. Y. Xu, K. T. Law, and P. A. Lee, Physical Review B **98** (2018), 10.1103/physrevb.98.121406.

[28] Y. Da Liao, Z. Y. Meng, and X. Y. Xu, Phys. Rev. Lett. **123**, 157601 (2019).

[29] R. Chitra and G. Kotliar, Phys. Rev. Lett. **84**, 3678 (2000).

[30] Y. Cao, V. Fatemi, A. Demir, S. Fang, S. L. Tomarken, J. Y. Luo, J. D. Sanchez-Yamagishi, K. Watanabe, T. Taniguchi, E. Kaxiras, and et al., Nature **556**, 80–84 (2018).

[31] A. Georges, G. Kotliar, W. Krauth, and M. J. Rozenberg, Rev. Mod. Phys. **68**, 13 (1996).

[32] H. Kajueter and G. Kotliar, Phys. Rev. Lett. **77**, 131 (1996).

[33] N. Dasari, W. R. Mondal, P. Zhang, J. Moreno, M. Jarrell, and N. S. Vidhyadhiraja, The European Physical Journal B **89** (2016), 10.1140/epjb/e2016-70133-4.

[34] M. Potthoff, T. Wegner, and W. Nolting, Phys. Rev. B **55**, 16132 (1997).

[35] G. Pálsson and G. Kotliar, Physical Review Letters **80**, 4775–4778 (1998).

[36] W. Xu, K. Haule, and G. Kotliar, Physical Review Letters **111** (2013), 10.1103/physrevlett.111.036401.

[37] P. Cha, A. A. Patel, E. Gull, and E.-A. Kim, "t-linear resistivity in models with local self-energy," (2019), arXiv:1910.07530 [cond-mat.str-el].

[38] E. H. Hwang and S. Das Sarma, Phys. Rev. B **79**, 165404 (2009).

[39] S. Das Sarma and E. H. Hwang, Phys. Rev. B **87**, 035415 (2013).

[40] M. Jonson and G. D. Mahan, Phys. Rev. B **21**, 4223 (1980).

[41] M. Jonson and G. D. Mahan, Phys. Rev. B **42**, 9350 (1990).

[42] M. R. Peterson and B. S. Shastry, Phys. Rev. B **82**, 195105 (2010).

[43] A. Kruchkov, A. A. Patel, P. Kim, and S. Sachdev, Phys. Rev. B **101**, 205148 (2020).

[44] J. Mravlje and A. Georges, Phys. Rev. Lett. **117**, 036401 (2016).

[45] R. A. Davison, W. Fu, A. Georges, Y. Gu, K. Jensen, and S. Sachdev, Phys. Rev. B **95**, 155131 (2017).

[46] S. Sachdev, Phys. Rev. X **5**, 041025 (2015).
